# Application of soil magnetometry and geochemical methods to investigate soil contamination with antimony

**DOI:** 10.1007/s10653-024-02086-0

**Published:** 2024-07-06

**Authors:** Magdalena Jabłońska-Czapla, Marzena Rachwał, Katarzyna Grygoyć, Małgorzata Wawer-Liszka

**Affiliations:** 1 Institute of Environmental Engineering of the Polish Academy of Sciences, 34 M. Skłodowska-Curie Street, 41-819 Zabrze, Poland; 2 Institute of Safety Engineering, Fire University, 52/54 Slowackiego St., 01-629 Warsaw, Poland

**Keywords:** Soil magnetometry, Soil contamination, PCA, PLI, WEEE, Traffic

## Abstract

The aim of the study was an assessment of the pollution level and identification of the antimony sources in soils in areas subjected to industrial anthropopressure from: transport, metallurgy and electrical waste recycling. The combination of soil magnetometry, chemical analyzes using atomic spectrometry (ICP-OES and ICP-MS), Sb fractionation analysis, statistical analysis (Pearson’s correlation matrix, factor analysis) as well as Geoaccumulation Index, Pollution Load Index, and Sb/As factor allowed not only the assessment of soil contamination degree, but also comprehensive identification of different Sb sources. The results indicate that the soil in the vicinity of the studied objects was characterized by high values of magnetic susceptibility and thus, high contents of potentially toxic elements. The most polluted area was in the vicinity of electrical waste processing plants. Research has shown that the impact of road traffic and wearing off brake blocks, i.e. traffic anthropopression in general, has little effect on the surrounding soil in terms of antimony content. Large amounts of Pb, Zn, As and Cd were found in the soil collected in the vicinity of the heap after the processing of zinc–lead ores, the average antimony (11.31 mg kg^−1^) content was lower in the vicinity of the heap than in the area around the electrical and electronic waste processing plant, but still very high. Antimony in the studied soils was demobilized and associated mainly with the residual fraction.

**Supplementary Information:**

The online version contains supplementary material available at 10.1007/s10653-024-02086-0.

## Introduction

Naturally, antimony (Sb) occurs as a trace element in soil and its main source is weathering of soil parent material. The enrichment of the soil/rock with antimony typically took place in the early stages of magmatic differentiation, and often occurs in hydrothermal metal sulfide deposits, either coprecipitated and associated with metal ores, or as separate mineral ores (Ashley et al., 2007).

Stibnite (Sb_2_S_3_) is the primary Sb ore, with valentinite (Sb_2_O_3_) widely occurring (Filella et al., 2002a, 2002b). Typical crustal abundances of Sb is reported as 0.2 mg kg^−1^ (Plant & Rainswell, 1983). Antimony concentrations are generally much lower than the corresponding values for As. According to Jones et al. (1990), the antimony content in soils of various countries ranged from 0.3 to 9.5 mg kg^−1^, while Wedepohl (1964–1979) indicates that the average for soils is 1 mg kg^−1^. In turn, the range of arsenic content in soils around the world ranges from 1 to 95 mg kg^−1^, and it is assumed that the average arsenic content in soils is calculated to be 4.4 mg kg^−1^ for podzols and 9.3 mg kg^−1^for histosols (Kabata-Pendias & Pendias, 1999). Antimony soil concentrations considered to be anthropogenic enrichment vary significantly due to differences in background concentrations and contamination limits between countries. Antimony soil concentrations have been comprehensively reviewed up to 2000 (Filella et al., 2009). The development of the mining and metallurgical, automotive and electrical industries, as well as the growing demand, rapid wear and “aging” of most products, including electrical/electronic equipment, necessitating their regular replacement, increases the amount of antimony in the environment. Anthropogenic sources of antimony are, for example: road traffic (Qi et al., 2011), recycling of electro-waste (Yurddaskal et al., 2018), as well metallurgy and mining (Azevedo et al., 2017; Bi et al., 2011; EU Council 1976; Guo et al., 2018; Perkins et al., 2014; Telford et al., 2008; Zeng et al., 2017). Sb contamination has most frequently been reported on and around mining and smelting sites often co-occurring with As (Ashley et al., 2007) and that relative concentrations of both metalloids depend on contamination source. Sb can be strongly retained in soils (Flynn et al., 2003; Wilson et al., 2010). Obviously, the extent of retention influences the bioavailable and mobile Sb fractions. Many factors impact retention, including soil characteristics and metalloid species present. Study of metalloid retention processes is fundamental for understanding biogeochemical cycling and for accurate risk assessment in different systems. Adsorption is one of the most important Sb retention mechanisms in soil (Bhattacharya et al., 2002; King, 1988). Oxides and hydroxides are known to be important for Sb adsorption in soil (Chen et al., 2003; Manaka, 2006; Mitsunobu et al., 2008). Antimony has been positively correlated with the soil iron oxide component (Denys et al., 2008). Therefore, the integrated geophysical-geochemical methods widely applied for assessment of soil quality with respect to trace element pollution (Magiera et al., 2006; Rachwał et al., 2017a, 2017b; Wawer et al., 2017) can also be used to estimate the location of a potential metalloid accumulation, including Sb.

Antimony is a toxic element, highly respirable, and its toxicity depends on many factors, including the degree of oxidation (Basel Convention, 2005; Bolan et al., 2022; Filella et al., 2009; Tschan et al., 2009). Trivalent antimony compounds are toxic to humans, mainly to the central nervous system and blood (Seńczuk, 1990; Yang & He, 2015).

The Upper Silesia is the highly urbanized and polluted region of Poland. An inactive metal-rich waste dumps are one of the most significant sources of heavy metal contamination, which emit pollutants (including PTEs) even over long distances (Jabłońska-Czapla et al., 2015; Rożek et al., 2015). The earlier studies indicated that soils in the surroundings of the metallurgical slag dump in Piekary Śląskie are characterized by high content of many elements (Cabała et al., 2008; Rachwał et al., 2017b; Rożek et al., 2015; Warchulski et al., 2015). Unfortunately, these studies did not focus on an element such as antimony (Sb).

The development of industry and communication infrastructure resulted in the emergence of many highways and expressways in Upper Silesia. Thousands of cars travel daily through these roads, which are also the source of metal and metalloid emissions to the environment. In road traffic, the main and the largest source of Sb contamination is car wearing parts such as car brake linings (0.07–201 mg kg^−1^ Sb) and car brake dust (4–16, 900 mg kg^−1^ Sb) (Kennedy, 2003a, 2003b; Qi et al., 2011 Thorpe & Harrison, 2008).

Electro-waste treatment plants (e-waste) are another source of antimony. Environmental contamination by e-waste recycling is an emerging global issue (Azevedo et al., 2017; Perkins et al., 2014). Compared to other types of waste produced in the world, the amount of e-waste is growing the fastest, both in terms of volume and environmental impact. Therefore it has gained significant popularity and scientific interest since 2010 (Kumar et al., 2017; Perkins et al., 2014. E-waste has attracted global concern owing to huge generation amount, rich valuable metal content, and potential environmental risk. When e-waste is recycled, Sb (an element being similar in chemical and physical properties to As) is released and contaminates the surrounding environment. Therefore, the ratio of Sb/As can be used for distinguishing the source of soil contamination, i.e. high Sb/As ratio may help identify the contamination due to the e-waste recycling activities (Bi et al., 2011).

The aim of the study was an assessment of the pollution level and identification of the antimony in soils in areas subjected to industrial anthropopressure from: transport, metallurgy and electrical waste recycling. An additional aim was to identify sites of potential accumulation of technogenic magnetic particles and accompanying PTEs, as well as antimony. The research attempted to use the soil magnetometry method in the quick estimation of soil contamination with PTEs. Magnetic susceptibility as the proxy tool, traditional geochemical analyses, sequential chemical fractionation analysis, as well as correlation and PCA analyses were applied in this research. While environmental indices (Pollution Load Index—PLI, Geoaccumulation Index—Igeo) as well as the Sb/As ratio (Bi et al., 2011; Fu et al., 2011; Sharifi et al., 2016 were computed in order to quantify the degree and origin of soil contamination by investigated elements. Compared to previous studies, this research provides more complex information about source-diversified soil contamination with antimony and other PTEs.

## Materials and methods

### Characteristics of study areas

**Fig. 1:**
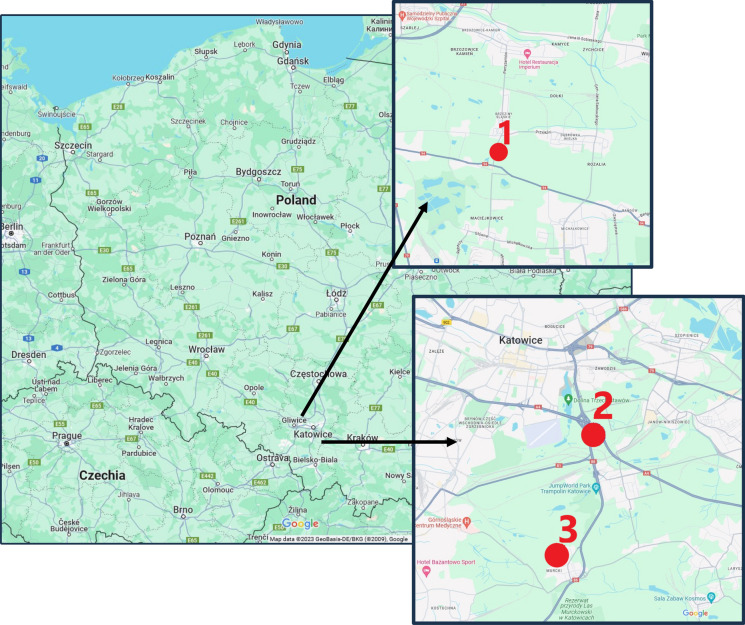
The location of the study areas 1. The area around the mining and smelting slag dump, 2. The area near highway, 3. The area around WEEE storage and processing plant

Three study areas were selected for the study: the area around the mining and smelting slag dump, the area around WEEE plant, and the area near highway. Figure 1 shows the location of the areas.

#### Electro-waste storage and processing plant

Soil samples were collected in the surrounding areas of WEEE (Waste Electric and Electronic Equipment) plant which was located in the Metropolis of Upper Silesia in Poland This region is dominated by luvisols made of boulder clays and clay and loam sands (Kondracki, 2002). Western winds dominate (approximately 60% of the share), and to a lesser extent eastern and southern winds and the average annual rainfall is 855 mm (Tokarska-Guzik et al., 2002). This area is heavily urbanized and is the most industrialized area of Poland, where numerous enterprises related to the mining and metallurgical industries are located. The company itself is located in the southern, heavily forested district of Katowice (Fig. 1). WEEE plant has been operating since 1995, and started processing waste electrical and electronic equipment in 2006. The company collects used devices and components from electrical and electronic devices. Additionally, the subject of interest of the company is any waste containing precious metals such as gold, silver, palladium, platinum, rhodium, and iridium. Waste is segregated and collected according to the type, stored in containers and big bags on a hard surface. The storage site is covered, which protects the waste from contact with rainfall and prevents the leakage of metal compounds and hazardous substances directly into the soil. In one of the closed halls, manual disassembly of electronic elements and components is carried out, and in another closed hall, the process of "skinning" of copper cables is carried out with the use of a cable recycling machine. So far, no metal/metalloid content studies have been conducted in the area around this plant.

#### Dump of slag remaining after Zn and Pb ore processing

Soils in the vicinity of the mining and smelting slag dump after Zn and Pb ores processing were selected for the research. This area consists of arable fields where vegetables are grown. The heap contains waste with a volume of approximately 3.3 × 106 m3 (mainly furnace slag with dolomite from rolling) from the former “Orzeł Biały" Mining and Smelting Company, which operated continuously for 150 years. The closed dump is located in the town of Piekary Śląskie (Upper Silesia, southern Poland; further in the text named just Piekary). In this region one of the largest resources of zinc and lead ores in Poland occur which are bounded to the Middle Triassic ore-bearing dolomites (Górecka, 1993). The soils in this area are rendzinas, formed on Triassic ore-bearing dolomites. The prevailing winds are west and north-west, the average annual rainfall is 817 mm.

The Piekary area has been monitored during recent years for several parameters, mainly heavy metals such as Pb, Zn, Cd, As (Kulka and Gzyl, 2008; Rachwał et al., 2017b; Ullrich et al., 1999; Warchulski et al., 2015), and only one work contained information, that antimony is present in the deposits in this area (Gałkiewicz & Śliwiński, 1983).

#### The area near a large communication junction

The third research area was the area located in Katowice in the vicinity of the A4 Motorway – the longest motorway in Poland, 672.75 km long, running from west to east through southern Poland. It belongs to the 3rd Pan-European Transport Corridor. Over one hundred thousand cars pass the A4 motorway in Katowice every day. Third research area is located in the Upper Silesian coal basin. The geological substrate here consists mainly of shales, sandstones and conglomerates containing hard coal deposits, and the prevailing soils in this area are luvisols (Kondracki, 2002). Due to high anthropogenic pressure, there are mainly anthropogenic soils composed of boulder clays. Characteristic here are weak winds, with a speed not exceeding 2 m/s, blowing from the west (Tokarska-Guzik et al., 2002). The research area includes a park near the A4 motorway in Katowice (Upper Silesia, Poland).

### Methodology

#### Magnetic investigations and sampling

Soil samples were collected around three above mentioned different sources of antimony emissions on the basis of the results of the preliminary in situ soil magnetic screening performed in accordance with the ISO Standard (ISO: 21226:2019, 2019). The magnetic susceptibility meter MS2 Bartington was equipped with MS2D loop sensor (Bartington Instruments Ltd., Witney, UK). At each individual point, the κ value was calculated as mean value of 11 readings within a 2m^2^ square. The κ value was expressed in 10^–5^ SI units. On the areas around the dump 26 points, around WEEE plant 19 points, near the highway 12 points, of surface magnetic susceptibility measurements were performed. Localization of individual points was confirmed using Garmin GPS navigation device. Based on the κ measurements, a maps of magnetic susceptibility distribution using Surfer 8 software (Golden Software Inc.) was prepared to select the most representative locations for soil sampling. Sampling was performed using soil sampler enabling collection of undisturbed 30-cm long soil cores (two from each place). Core samples were taken into plastic tubes at different distances from the pollution source. Due to the fact that it was not possible to collect soil cores in the vicinity of the motorway, soil samples were collected in bags using a plastic spatula. The soil was heavily transformed there, usually it contained road construction elements, and it was impossible to drive a soil probe there.

After transporting soil cores to the laboratory, in order to determine the vertical distribution of magnetic susceptibility and indirectly the depth of migration of pollutants along the soil cores, the magnetic susceptibility was measured using the Bartington MS2C meter, with a resolution of 1 cm. From the depths characterized by the highest values of magnetic susceptibility (mostly at the depth of 3–6 cm), soil samples were separated and subjected to chemical analysis after air drying (at 21 °C), averaging and sieving through a sieve with a diameter of 2 mm. Before chemical treatment, the volume magnetic susceptibility (κ) of the samples was measured using a Bartington MS2B sensor (Bartington Instruments Ltd., Witney, UK) and the corresponding mass-specific susceptibility (χ) was calculated according to the International ISO Standard (ISO 21226:2019, 2019). Samples taken from the depth at which the highest magnetic susceptibility value occurred in the core were further analyzed.

### Chemical analyses

#### Basic physicochemical parameters

Afterwards, the pH and Eh of the soils were measured using the multifunction meter CX-401 (Elmetron, Poland) in accordance with the standards (PN-ISO 10390:1997). An ERH-111 electrode (Elmetron, Poland) was used for pH measurements, while Eh were measured using ERPt-111 electrode (Elmetron, Poland).

#### Digestion and determination of metals and metalloids in soil samples

In order to determine the total element content, soil samples were digested in a microwave oven (Microwave 3000, Anton Paar). 0.2 g of soil was prepared and digested with 5 ml of HNO_3_ (spectral purity, Merck, Germany), 2 ml of H_2_O_2_ and 3 ml of HF (spectral purity, Merck, Germany). Digestion program: 1400 W, 35 min. After microwave digestion samples were diluted to 50 ml in PP flask. Afterwards, they were stored in a fridge at 2–5 °C. Each sample was measured three times using ICP-MS or ICP-OES.

Elements such as As, Mo, Cd, Co, Tl and Sb were determined with an ICP-MS Elan DRC-e 6100 spectrometer (Perkin Elmer). Apparatus was equipped with a standard ICP torch, cross-flow nebulizer and nickel cones. Samples and standards were delivered with a peristaltic pump. The spectrometer was optimized daily with a 10 μg^.^L^−1^ solution (Mg, Cu, Rh, Cd, In, Ba, Ce, Pb, U) in 1% HNO_3_ Elan 6100 Setup/Stab./Masscal. Solution (Perkin-Elmer). Concentrations of ^59^Co,^75^As, ^98^Mo, ^114^Cd, ^205^Tl and ^123^Sb were measured with the internal ^103^Rh standard.

Elements such as Ba, Cr, Cu, Pb, Sr, Zn, Al and Fe were analyzed using Avio 200 ICP-OES spectrometer. Apparatus was equipped with a standard ICP torch, corundum nozzle, cross-flow nebulizer and Scott fog chamber. The spectrometer was optimized daily using the Optima Family Multi-Element Standard optimization solution (Perkin Elmer). The operating parameters of the ICP-MS and ICP-OES spectrometer are presented in Supplementary Information (Table S1).

#### Reagents

The following substances were used for ICP-MS analysis: Mix 1 (Sigma-Aldrich, Switzerland), Metalloid and non-metal mix (Sigma-Aldrich, Switzerland), Rhodium standard (Merck, Germany), ICP multi-element standard XVI (Merck, Germany). Multi Element ICP Standard solution (Chem-Lab, Belgium) and ICP multi-element standard solution IV (Merck, Germany) were used for ICP-OES analysis. Working standard solutions were obtained by appropriate dilution of the stock standard solutions using Milli-Q-Gradient ultra-pure deionized water (Millipore, Milli-Q-Gradient ZMQ5V001). The Merck suprapure (Germany) 65% nitric acid was used for preparing samples, blanks and for apparatus rinsing. Hydrogen peroxide 30% for analysis (Merck, Germany), hydrofluoric acid 40% spectral purity (Merck, Germany) and nitric acid 65% suprapure (Merck, Germany) were used for digestion soil samples.

#### Quality control

The analysis of real samples was preceded by the preparation of a calibration curve (blank sample solution and certified standard solutions of known concentration). As part of quality control, a calibration curve was checked. After calibration and once every 5 real samples, a blank sample and control solutions with known concentrations of each analyte were analyzed to monitor cross-contamination. While the measured concentration of elements has changed by more than 10%, recalibration and repeated sample analysis were performed. Independently of the calibration solutions and to check the calibration, control samples with concentrations from the lower and upper parts of the curve were prepared. The results obtained were plotted on a control chart to monitor warning values. During each measurement series, a repeated sample was analyzed to verify the precision of preparation and analysis. Additionally, the standard deviation was monitored by three times measurement of each sample. To verify the methodology for determining the total metal/metalloid content, digestion, and analysis of the certified soil reference material NCS DC 73324 (China National Analysis Center for Iron and Steel) was carried out under the same conditions as other soil samples. Table 1 shows the recovery of the tested metals and metalloids in CRMs (Certified Reference Material) and the basic validation parameters for the determination of elements using the ICP-OES and ICP-MS techniques. During each digestion, a blank (reagents without soil) sample was prepared to monitor accidental sample contamination during sample preparation.

**Table 1:** Basic validation parameters and soil Certified Reference Material NCS DC 73324 recovery

Analyte	Isotope/spectral line [nm]	LOD [mg kg^−^]	LOQ [mg kg^−^]	Soil CRM values from the certificate [mg kg^−1^]	Measured soil CRM values [mg kg^−1^]	Recovery [%]
As	75	0.03	0.13	220 ± 14	222	101
Tl	205	0.05	0.15	2.4 ± 0.5	2.7	111
Mo	98	0.05	0.15	18 ± 2	19.1	106
Cd	114	0.02	0.07	0.13 ± 0.03	0.12	94
Co	98	0.002	0.014	7.6 ± 1.1	7.4	98
Sb	121	0.05	0.15	60 ± 7	62.7	104
Cr	205.560	2.0	6.0	75 ± 6	75.5	101
Pb	217.000	2.0	6.0	314 ± 13	341	108
Zn	213.857	2.0	6.0	97 ± 6	98.9	102
Cu	327.393	2.0	6.0	390 ± 14	455	116
Fe	239.562	1.8	5.3	56,582 ± 909	52,267	92
Al	394.401	10	2.5	212,300 ± 846	2,000,750	94
Sr	421.552	2.0	6.0	39 ± 4	38.2	98
Ba	233.527	2.0	6.0	118 ± 14	125	106

*LOD* limit of detection, *LOQ* limit of quantification, *CRM* certified reference material, LOQ = 3*LOD

#### Sequential chemical extraction

The modified BCR (the Institute for Reference Materials and Measurements) sequential chemical extraction helped to determine the antimony forms in soil and the way in which they were bound. Sequential chemical extraction included stages: F0 mobile dissolved antimony in water, F1 mobile exchangeable fraction associated with adsorbed cations and anions, carbonates, and very reactive oxy-hydroxides; F2 mobile reducible fraction associated with iron/manganese oxides; F3 immobile oxidisable fraction associated with organic substance and sulfides; F4 (R) immobile residual fraction associated with non-silicate bound metals. The extraction methodology has been described previously in the paper (Larner et al., 2006).

#### Calculation and data analysis

The obtained results were statistically analyzed in Excel and Statistica 12. In order to better interpret the obtained results, the correlation coefficients in the form of a correlation matrix were calculated and the principal components analysis (PCA) was performed. Calculation of complex linear correlations between content of all elements was carried out by PCA. Afterwards, as a result of orthogonal varimax rotation, factor loadings of principal components were extracted (using their content in the soil of the studied objects). For each research area, two principal components, responsible for the cumulative variance from PCA were extracted.The evaluation of soil pollution was performed using Sb/As factor, which is one of the indicators of pollution sources (Bi et al., 2011; Fu et al., 2011), Geoaccumulation Index (I_geo_) proposed by Müller (Müller, 1969, 1979) and Pollution Load Index (Tomlinson, 1980).

Geoaccumulation Index (Igeo) is defined as:

$${\text{I}}_{{{\text{geo}}}} = {\text{ log}}_{{2}} \left( {{\text{C}}_{{{\text{EL}}}} /{1}.{\text{5 C}}_{{{\text{background}}}} } \right),{\text{ where}}$$

C_EL_ is total element concentration in soil samples, C_background_ is the geochemical background of element concentration, factor 1.5 is a correction factor compensating for natural (lithological) variations in geochemical data. I_geo_ describes the pollution of soil by an element with respect to seven classes from 0 to 6. To the class 0 (I_geo_ ≤ 0), belong non-polluted soils. First class (0 < I_geo_ ≤ 1) describes uncontaminated to the moderately contaminated soils. Second class (1 < I_geo_ ≤ 2) is moderately contaminated soils. The third class (2 < I_geo_ < 3) concerns soils moderately or heavily polluted. The heavily contaminated soils are included in the fourth class (3 < I_geo_ < 4). Then to the fifth class (4 < I_geo_ < 5) belong heavily to very heavily polluted soils. And the last one is sixth class (I_geo_ > 5) with very contaminated soils.

Pollution Load Index (PLI) is often used to determine the level of soils contamination compared to background concentrations levels (Tomlinson, 1980). To calculate the PLI, the following equations were used:

$${\text{CF}}_{{{\text{EL}}}} = {\text{ C}}_{{{\text{EL}}}} /{\text{C}}_{{{\text{background}}}}$$

$${\text{PLI }} = \, \left( {{\text{CF}}_{{{\text{EL1}}}} \times {\text{CF}}_{{{\text{EL2}}}} \times {\text{CF}}_{{{\text{EL3}}}} \times \ldots \ldots \times {\text{cF}}_{{{\text{ELn}}}} } \right)^{{{1}/{\text{n}}}}$$

where CF_EL_—the pollution factor defined as the ratio of the element concentration in the analyzed soil sample to the concentration of this element in the background.

In order to compute the above-mentioned indices, the following geochemical background values (in mg kg^−1^) were adopted after Geochemical Atlas of Europe (de Vos et al., 2006): As—11.6; Tl—0.821; Mo—0.943; Cd—0.284; Co—10.4; Sb—1.04; Ba—400; Cr—94.8; Cu—17.3; Mn—524; Pb—32.6; Sr—130; and Zn—68.1.

## Results and discussion

### Magnetic properties of soils from studied area

**Fig. 2:**
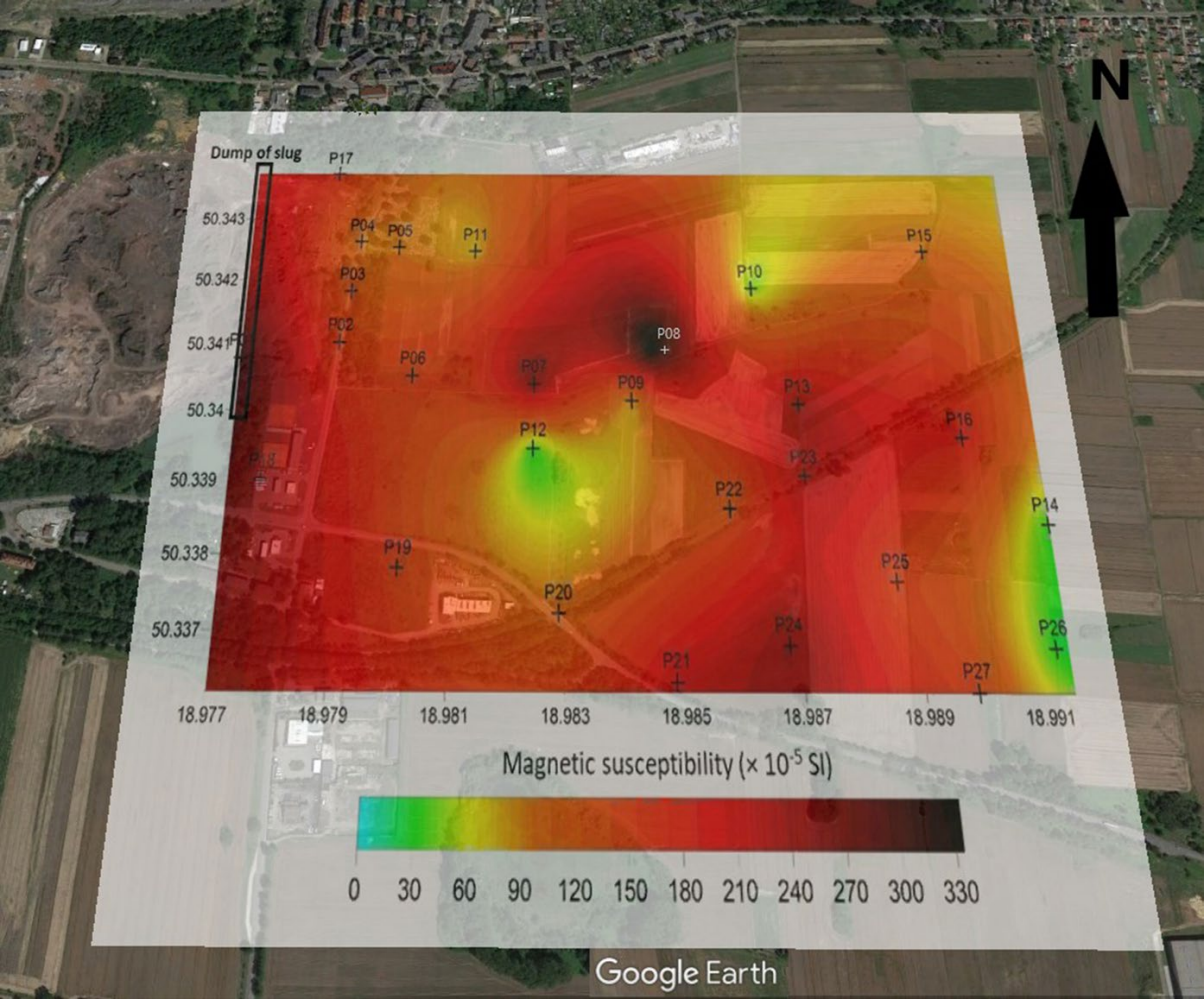
Spatial distribution of soil magnetic susceptibility in the area of the impact of the mining and smelting slag dump. Geographic coordinates are located at the edges of maps (source of the base map: Google Earth)

**Fig. 3:**
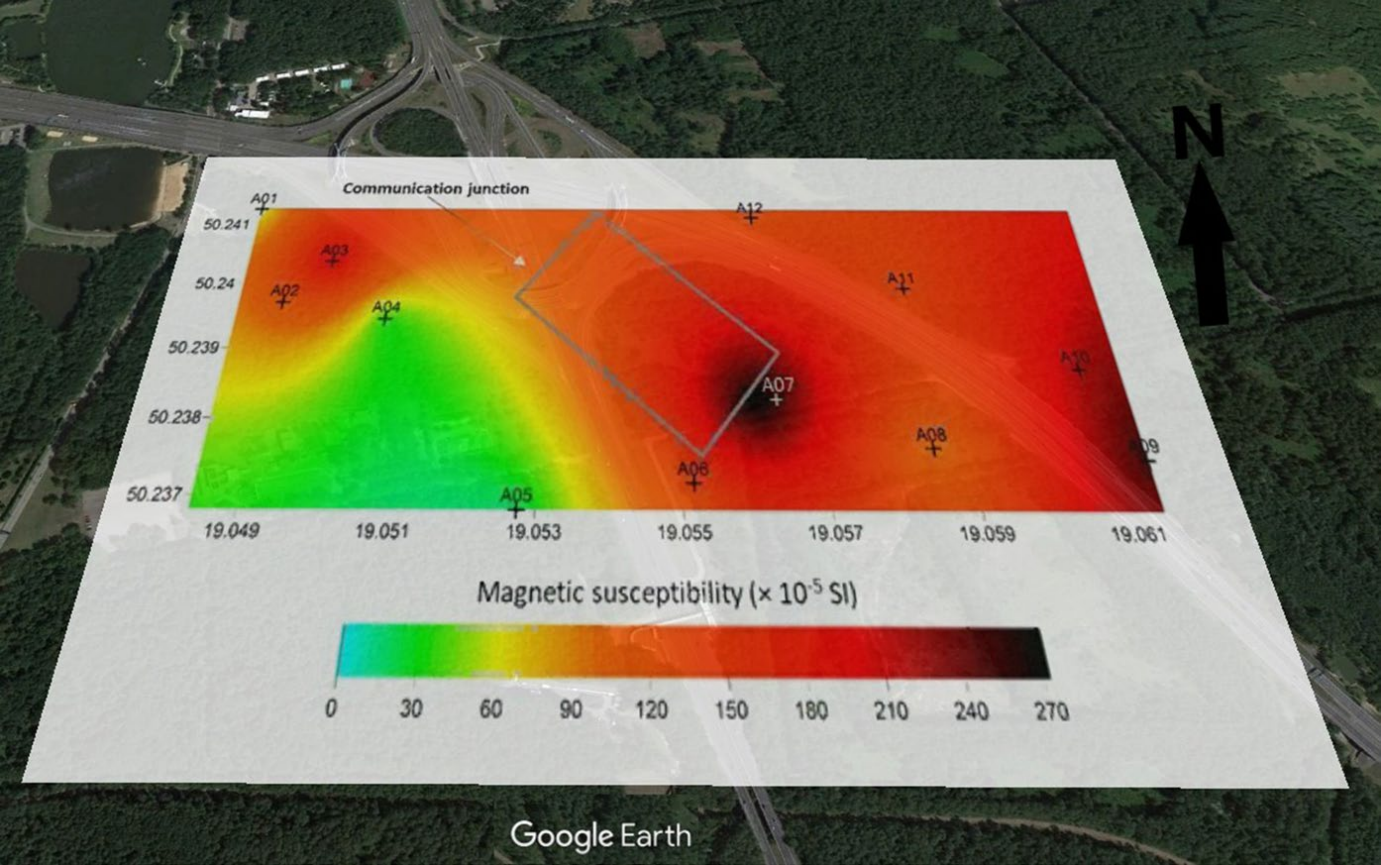
Spatial distribution of soil magnetic susceptibility in the highway area. Geographic coordinates are located at the edges of maps (source of the base map: Google Earth)

**Fig. 4:**
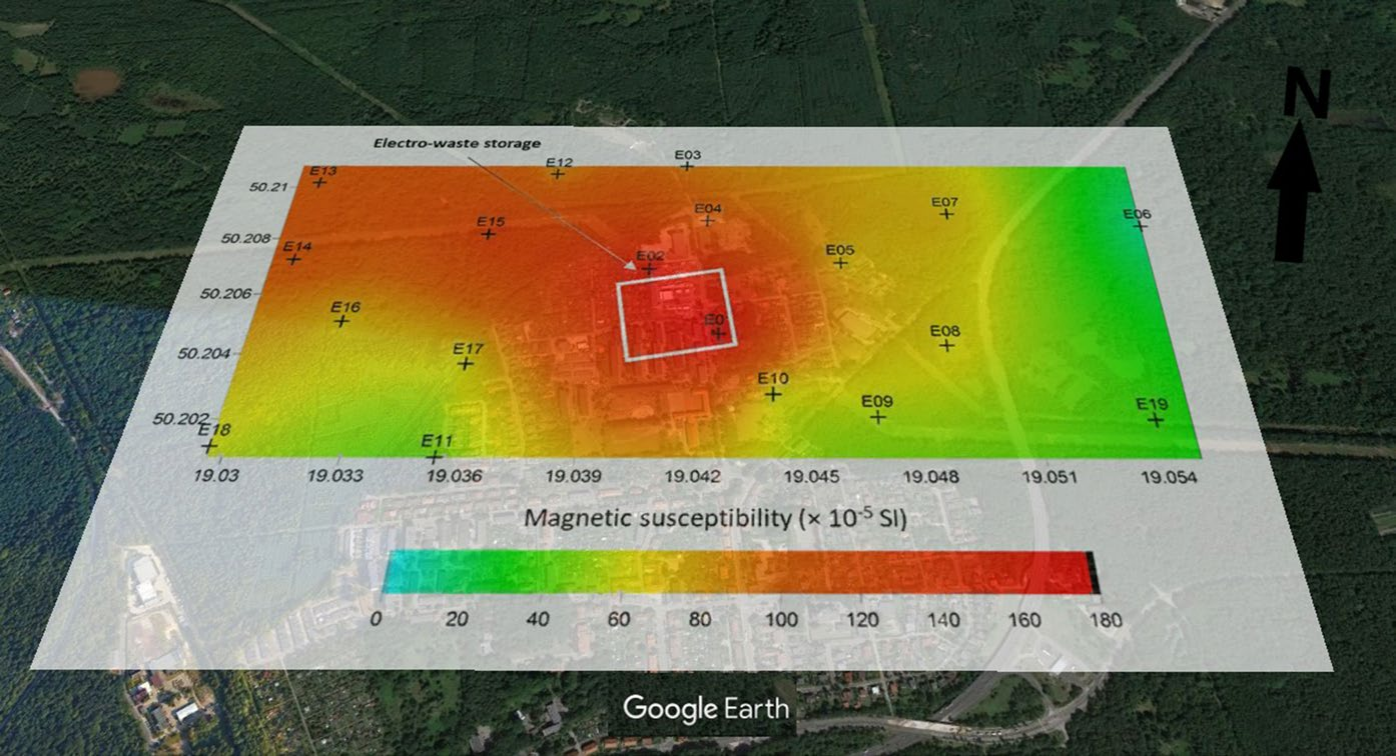
Spatial distribution of soil magnetic susceptibility in the area affected by the electrical and electronic waste processing plant. Geographic coordinates are located at the edges of maps (source of the base map: Google Earth)

A large diversification of the degree of soil contamination in the area impacted by various sources was stated. The magnetic susceptibility values measured in the field (κ) show that the most contaminated soils are found in the area of the mining and metallurgical slag dump and motorway (up to 330 and 270 × 10^–5^ SI, respectively), and the lowest in the area of the electrical and electronic waste treatment plant (up to 170 × 10^–5^ SI) (Figs. 2, 3, and 4). Compared to magnetic background of the soils (26–50 × 10^–5^ SI; Rachwał et al., 2017b), the tested soils with much higher values of κ can be considered heavily contaminated with the technogenic magnetic particles resulting from the various technological processes and combustion of solid fuels (Catinon, et al., 2014; Magiera et al., 2011). The points with the lowest ĸ values are located in wooded areas (surroundings of WEEE plant), where soil profile is undisturbed. Much higher κ values were observed in places heavily transformed by human activity, i.e. area around dump with fields fertilized with sewage sludge and surfaces polluted by resuspended metallurgical dusts or area near highway regularly contaminated by traffic. The soil in these places is a typical technosol with numerous artifacts in the form of pieces of asphalt, bricks or debris.

However, in all tested objects, the magnetic susceptibility values decrease with the distance from the source of emission of technogenic magnetic particles (dump, WEEE plant, highway), but the spatial distribution of κ indicates the presence of numerous “hot spots”, i.e. points with higher κ values. In the case of metallurgical dump, higher values of κ were observed not only in the vicinity of the dump, but also in the fields fertilized with sewage sludge (Fig. 2). Around the electrical and electronic waste processing plant (Fig. 4), high κ values in the north-west part of the study area are due to the impact of low emissions from domestic furnaces (low-rise single- and multi-family buildings).

#### Dump area

**Fig. 5:**
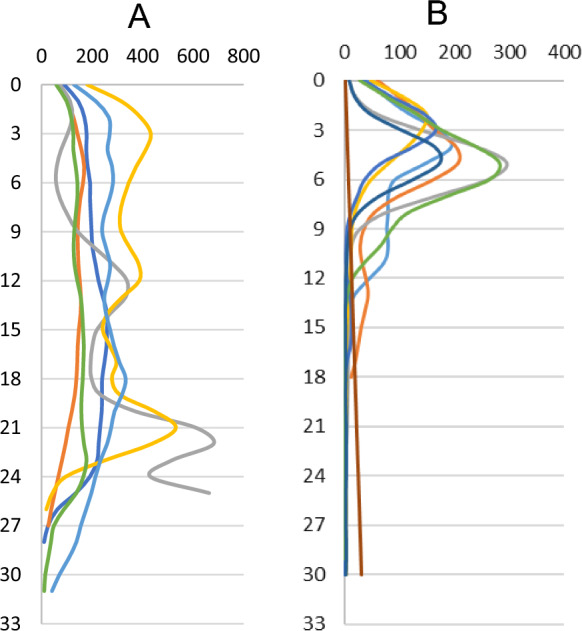
Vertical distribution of magnetic susceptibility (ĸ) in soil profiles from: A the area of the impact of the mining and smelting slag dump and B WEEE plant (ordinate axis: depth in cm; abscissa: ĸ values (× 10^–5^ SI)

The cores collected in the cultivated area in the vicinity of the dump were characterized by a disturbed profile associated with plowing. Particular soil levels are disturbed and the magnetic signal has been diluted due to the mixing of the arable soil layer with the lower soil horizons as a result of agrotechnical treatments (Magiera et al., 2006). Despite these treatments, the susceptibility values in these cores were high (from 50 to over 300 × 10^–5^ SI) (Fig. 5).

Mass-specific magnetic susceptibility values of individual samples cut from soils cores collected in the dump area varied from 62 to 308 × 10^−8^ m^3^ kg^−1^ (average 212 × 10^−8^ m^3^ kg^−1^) (Table 2). The region of Piekary has been a place of mining and processing of zinc–lead ores for centuries, therefore it is strongly transformed by human activity. The contamination of the soil in this place is caused not only by the blowing and washing of material from the heap, but also by the use of sewage sludge to fertilize the field (Rachwał et al., 2017b). Sewage sludge is rich in metals and metalloids and therefore their use increases accumulations of Cd, Cr and Pb in soil (Singh & Agrawal, 2008; Urionabarrenetxea et al., 2022).

**Table 2:** The results of total elements concentration as well as magnetic susceptibility (χ) in selected soil samples from the dump area

	χ	As	Tl	Mo	Cd	Co	Sb	Ba	Cr	Cu	Mn	Pb	Sr	Zn	Al	Fe
unit	10^–8^ m^3^ kg^−1^		mg kg^−1^												g kg^−1^	
P02	198	30.48	2.14	0.89	23.32	5.76	7.02	285	54.41	38.47	625	805	47.50	2157	14.76	19.47
P06	308	39.93	1.82	1.29	12.59	7.92	21.32	195	55.79	84.82	723	1054	40.17	1736	12.53	24.67
P07	268	28.08	0.72	0.81	8.38	5.90	2.67	299	40.21	21.85	571	294	51.49	1022	6.29	16.68
P08	249	51.36	1.46	1.15	16.59	7.91	4.82	221	58.73	90.05	400	699	39.23	1835	13.36	20.28
P11	252	73.69	2.11	1.27	24.74	9.39	6.15	131	49.23	75.64	852	698	35.52	2298	9.46	26.45
P12	62	11.37	0.97	0.65	8.07	5.14	12.14	210	41.73	24.49	342	435	37.96	681	15.28	12.89
P14	117	14.94	1.45	1.67	10.94	7.82	3.01	270	52.32	22.98	757	400	54.88	1156	16.48	17.67
P17	242	57.22	1.29	1.07	4.85	9.35	33.35	492	26.71	54.58	534	1220	194	1323	3.12	11.65
Mean	212	38.38	1.50	1.10	13.69	7.40	11.31	263	47.39	51.61	601	701	62.55	1526	11.41	18.72

#### WEEE area

In the case of the area affected by the electrical and electronic waste processing plant, the cores were collected mostly in wooded areas, which is reflected in the vertical distribution of magnetic susceptibility, the highest values of which are in the top organic soil horizons (3–5 cm), and then they drop even to values close to zero down to a depth of 30 cm. Such a vertical distribution of the κ value indicates a high concentration of dust pollutants in the topsoil (Blaha et al., 2008; Fialová et al., 2006).

The samples from the WEEE region had significantly higher values of mass-specific magnetic susceptibility—it ranged from 292 to 862 × 10^−8^ m^3^ kg^−1^ (average 681 × 10^−8^ m^3^ kg^−1^) (Table 3). Previous studies conducted in this area by Łukasik et al. (2015) revealed lower values of the magnetic susceptibility of soils (340 × 10^−8^ m^3^ kg^−1^ on average), which indicates the direct impact of the WEEE company on pollution.

**Table 3:** The results of total elements concentration as well as magnetic susceptibility (χ) in selected soil samples from the area of the electrical and electronic waste processing plant

	χ	As	Tl	Mo	Cd	Co	Sb	Ba	Cr	Cu	Mn	Pb	Sr	Zn	Al	Fe
Unit	10^–8^ m^3^ kg^−1^		mg kg^−1^												g kg^−1^	
E02	862	70.17	3.27	6.41	7.43	21.61	27.39	455	128	91.51	630	1476	46.70	631	14.38	37.26
E04	804	62.03	3.72	4.99	4.66	14.23	14.47	299	88.38	92.66	301	1435	42.81	732	16.61	34.53
E05	685	57.34	2.54	4.64	5.58	15.68	16.57	428	117	92.47	594	1369	44.61	593	14.60	32.07
E07	292	13.43	1.95	2.24	2.69	6.24	6.58	187	52.19	21.40	140	434	32.53	239	15.23	14.07
E09	514	50.32	2.66	3.69	3.90	12.76	12.84	358	77.76	60.70	291	809	34.03	349	12.82	27.16
E13	716	55.40	2.96	5.85	6.10	16.72	20.78	555	104	110	299	1424	53.15	522	16.21	38.35
E15	751	57.67	2.19	7.66	35.93	17.23	17.15	234	132	111	252	1319	32.01	413	15.22	35.81
E17	824	50.67	2.70	5.43	3.47	21.26	14.64	343	124	78.05	251	665	43.23	413	14.41	33.35
Mean	681	52.13	2.75	5.11	8.72	15.72	16.30	358	102	82.18	345	1116	41.13	487	14.94	31.58

#### Highway area

Unfortunately, it was not possible to collect soil cores in the vicinity of the motorway. The land there has been heavily transformed, the soil is poorly developed, being rather a mixture of soil and road construction materials, therefore soil samples were collected in bags using a plastic spatula.

Surprisingly, the lowest average value of χ was observed for soil in the vicinity of the motorway: 195.7 × 10^−8^ m^3^ kg^−1^ (min. 12 and max. 419 × 10^−8^ m^3^ kg^−1^) (Table 4). Still, these values were higher than the background values for this region (approx. 50 × 10^−8^ m^3^ kg^−1^, Jordanova, 2016). Similar values in the vicinity of the roads of Upper Silesia were obtained by Wawer et al., (2015, 2017). These data prove a significant contamination of soils from the studied areas with technogenic magnetic particles, which are good carriers of potentially toxic elements (Magiera et al., 2013; Rachwał et al., 2015).

**Table 4:** The results of total elements concentration as well as magnetic susceptibility (χ) in selected soil samples from the highway area

	χ	As	Tl	Mo	Cd	Co	Sb	Ba	Cr	Cu	Mn	Pb	Sr	Zn	Al	Fe
unit	10^–8^ m^3^ kg^−1^		mg kg^−1^												g kg^−1^	
A01	12	6.74	0.54	0.92	1.45	6.03	1.06	130	46.69	20.06	655	75	32.42	288	9.07	13.34
A03	255	25.11	0.92	1.74	6.52	13.64	3.00	260	96.58	60.33	896	825	57.01	3000	20.11	27.51
A04	58	2.98	0.30	0.69	0.72	2.17	0.69	177	26.94	7.26	202	23.91	24	95	8.63	4.86
A06	136	28.52	1.23	3.52	5.55	11.15	2.33	247	68.26	40.86	403	270	38.97	1205	11.08	18.41
A07	419	40.52	1.17	1.60	8.34	17.14	9.98	385	83.71	77.63	1016	567	88.05	2315	17.72	27.73
A08	72	37.77	0.90	1.74	2.86	11.83	6.61	146	51.80	69.24	341	525	32.97	1497	11.44	15.10
A09	330	40.06	0.85	3.92	5.58	12.43	5.7	218	127	72.99	1016	471	55.80	1992	15.61	24.78
A11	170	11.59	0.73	0.97	3.09	6.47	2.13	181	62.36	29.77	454	244	38.68	823	14.38	15.09
Mean	195.7	24.16	0.83	1.89	4.26	10.11	3.88	218	70.47	47.27	623	375	46.02	1402	13.51	18.35

### Total antimony and other elements in soils from studied area

#### Dump area

In all tested soil samples in the vicinity of the dump, the antimony content was high and amounted to an average of 11.31 mg kg^−1^. The highest concentration of antimony amounted to 33.35 mg kg^−1^ was found closest to the heap in the soil sample P17 (Table 2). In samples taken in the nearby dump, a high content of this element was found in sample P06, amounting to 21.32 mg kg^−1^. As expected, very high amounts of lead (up to 1220 mg kg^−1^ in the P17 sample) and zinc (up to 2298 mg kg^−1^ in the P11 sample) were found in all soil samples collected in the vicinity of the dump. In the case of elements such as Cr, Mn, Fe, Mo, Tl in all soil samples in this area, the content of these metals differed slightly. With regard to the limit values regulated by Polish legislation, the soils in the area surrounding the dump in Piekary are heavily contaminated with Cd, Zn, Pb (Regulation of the Minister of the Environment, 2016). The sampling area was located east from the dump in the prevailing wind direction. The Polish legislation does not specify the maximum content of antimony in soil. The antimony content in most of Polish soils reaches a maximum of 0.52 mg kg^−1^, and on average it amounts to 0.17 mg kg^−1^ in the areas of permanent grassland (Pasieczna, 2012. Even in the areas enriched with antimony such as the Pszczyna region, the content of this element did not exceed 3 mg kg^−1^ (Loska et al., 2004). The antimony content in the research areas significantly differs from those naturally occurring of geogenic origin.

Literature data indicated that anthropogenic activity may cause a significant increase in antimony contamination of soil. For example, in the area affected by the lead and zinc smelter, the antimony concentration in the urban topsoil was recorded at the level of 2.5–175 mg kg^−1^ (Douay et al., 2008), while in the area polluted by mining, the concentration of antimony ranged from 26 to 1150 mg kg^−1^ (Denys et al., 2008). Qi et al. (2011), examining soil samples collected from a coal mine area in Anhui Province, China, found that more than 75% of soil samples showed a significant degree of Sb contamination, with an average Sb concentration of mg kg^−1^. It is disturbing that the object of the presented research in the vicinity of the heap was arable soil, and it turns out that long-term fertilization may increase the content of potentially toxic elements in the soil, including antimony (Leita et al., 1999).

#### WEEE area

Large amounts of antimony were also found in the soils around the WEEE plant (Table 3). The highest content of 27.39 mg kg^−1^ was found in sample E02, located in the immediate vicinity of this plant. Taking into account all research objects, it was stated that the WEEE plant has the utmost effect on the antimony content in the soil. In its vicinity, the average Sb content in the soil was as much as 16.30 mg kg^−1^ (for comparison: the average Sb content in the area of the heap and the highway was 11.31 and 3.88 mg kg^−1^, respectively).

As in the soils of the area surrounding the dump, high amounts of antimony were found in the soils around the WEEE plant (Table 3). In sample no. E02, located in the immediate vicinity of this plant, the Sb content was even 27.39 mg kg^−1^. Taking into account all the results of the quantitative analysis of antimony in the tested soil samples, it turns out that the WEEE processing and sorting plant has the greatest impact on the content of antimony in the soil. In the vicinity of this plant, the average concentration of Sb in the soil was as high as 16.3 mg kg^−1^. Our results confirm previous studies conducted by Quan et al. (2015), which stated that Sb concentration in soil around e-waste processing plant, was the highest in surface and middle soil, with Sb concentration of 16.3 and 20.2 mg kg^−1^. Similar results were obtained by Bi et al., (2011). In addition to the high antimony content in the soil, the e-waste processing and sorting plant is a source of metals such as lead, manganese and cadmium. The concentration of lead in the soils surrounding this source even exceeded the content of this element in the soils in the area under the pressure of the dump in Piekary. The content of lead in the topsoil was even 1476 mg kg^−1^ Pb in sample E02 (closest to the plant). At this point, apart from lead, there were high contents of Cd, Mn, Co. The high lead content in the soils of this place results not only from emissions from the neighboring heap, but is also related to the local geological structure and the occurrence of Triassic ore dolomites (Cabała et al., 2008; Rachwał et al., 2017b).

#### Highway area

Increased car traffic causes additional pollutants to appear in the environment, which, when removed from the road surface by wind or rainfall, accumulate in the soil. Automotive brake pads can be a serious threat to the environment. During braking, the brake pads are worn down, which, combined with the high temperature, can release the brake pads material. Sb_2_S_3_, is a solid lubricant, plays an important role in brake performance and can react, oxidize or decompose with exposure to high temperatures (Martinez & Escheberria, 2016).

In the case of the research area near the A4 motorway, the highest concentration of antimony was found in the soil sample number A07 and it was 9.98 mg kg^−1^ Sb, but the mean concentration of antimony in this area was below 10 mg kg^−1^, average 3.88 mg kg^−1^ (Table 4). This is the lowest content compared to other research areas. This means that road traffic and brake pad wear have little effect on the antimony content in roadside soil. Unfortunately, research has shown that car traffic has a significant impact on the increase in the content of manganese and zinc in the soil which was comparable to the concentration of these elements around the dump in Piekary. Interestingly, the concentration of lead in soils surrounding the expressway is high, averaging 375 mg kg^−1^, despite the fact that lead-containing fuels have not been used for almost 20 years. The increased concentration of manganese in soils in the area surrounding the A4 motorway is most likely related to the abrasion of the road surface during car traffic. In turn, high zinc content is associated with corrosion of galvanized panels, safety barriers or other elements constituting the infrastructure of the motorway. This phenomenon has been confirmed previously by Hjortenkrans et al. (2007), Świetlik et al. (2013 or Wawer et al. (2017).

### Mobility of antimony in soil in areas of varying anthropopressure

Table S2 (Supplementary Information) shows the results of pH and Eh analyzes of soils collected from three study areas. In order to determine the mobility of antimony, six soil samples were selected from each area and tested for pH and Eh.

As shown in Fig. S1 (Supplementary Information), antimony in the soils in the study areas was associated mainly with the residual fraction. In all soil samples, the residual fraction had the largest share. In the samples of soils collected around the dump and around the motorway, there was a greater proportion of antimony in the F0 (dissolved in pore water) and F1 (ion exchange and carbonate) fractions, compared to the soils collected in the areas affected by the e-waste processing plant. The highest share of antimony in the ion exchange and carbonate fractions was found in the A11 soil sample collected at the A4 motorway. In turn, the share of antimony in the oxide fraction (F2) was significantly higher in the soils subjected to the pressure of the WEEE plant. The soils around the WEEE plant, as shown in Table S2 (Supplementary Information), are characterized by different physicochemical parameters. The soils in these areas have a lower pH (from 3.35 to 4.33) and a higher redox potential (from 349.3 to 425.5 mV) compared to the soils collected from the areas around the dump and the motorway. The transformations of many soil components and the introduced pollutants are related to redox transformations. By determining the redox potential in soil, its oxygen state can be determined. The value of + 300 mV has been proposed as the boundary between oxidation and soil reduction conditions (Stępniewska et al., 2004). The redox potential expresses the oxidation–reduction state of the soil, which is a measure of the system's reduction. It presents the proportions between the oxidized and reduced forms of elements present in the soil and the resilience of free electrons displaced in redox reactions taking place under various oxygenation conditions. With its help, it is possible to determine the rate of oxidation–reduction changes taking place and the time after which anaerobic conditions begin to appear in excessively moist soils and the reduction process begins. The value of + 200 mV was assumed as this limit. A higher potential occurs when the soil is dominated by electron acceptors, creating a positive potential, e.g. O_2_, NO_3_, MnO_2_, Fe_2_O_3_. The limiting value is the potential of + 300 mV, which corresponds to the reduction of iron (III) to iron (II). Other boundaries of the ranges divide the redox potential that characterizes soil conditions into three ranges: over + 300 mV (oxygenated soils), from + 300 to –100 mV (processes of reduction of oxygen connections of nitrogen, manganese and iron–soils that are slightly and moderately reduced), and from − 100 to − 300 mV (sulphate reduction processes and subsequent methane formation—reduced soils). On this basis, it can be concluded that in the soils from the areas around the dump and the motorway, the processes of reducing oxygen connections of nitrogen, manganese and iron prevail (soils with low and moderate reduction). On the other hand, the soils collected from the areas surrounding the electrowaste treatment plant are characterized by higher Eh (over 300 mV) and they are oxygenated soils. Depending on origin, the relationship of antimony with silicates can be very important. In many natural environments, adsorption of Mn and Fe oxides and hydroxides is responsible for retaining antimony in the soil. The extent of antimony retention in soil may be smaller or greater than the analogous behavior of arsenic in this environment, depending on the forms and sorption surface present. This has an impact on the bioavailability and mobility of antimony. The mechanisms by which Sb interacts with the various phases are still largely unknown as the effects of protonation and specific adsorption of antimony in acidic soils have not been studied so far. Hence, it is important and necessary to study the mobility of antimony in soils of various origins and the impact of human activity. There are general trends in Sb fractionation in soil. These include low mobility of Sb and a high proportion of Sb associated with the oxyhydroxide containing fraction of the soil or the residual fraction depending on the source of the metalloid. It is known that the metalloid associated with the crystalline and non-crystalline oxide and hydroxide minerals is immobile (Tighe & Lockwood, 2007). Nevertheless, desorption and/or reductive dissolution of the oxygen-hydroxide phase of soil may occur as a result of redox changes under various environmental conditions (Filella et al., 2002b). This has important implications for the potential availability and mobility of metalloids under certain environmental conditions.

### Assessment of soil contamination

The conducted research indicate a wide variation in the degree of soil contamination in areas affected by various facilities on the environment. All studied areas are characterized by excessive metal/metalloid content in the soil. Aluminum and iron have the highest levels in the soils of the studied areas, but they are major elements, so their amounts will not be considered as contamination. On the other hand, elements such as As, Cd, Pb and Zn, considered as substances causing risk, accumulated in the soil to a degree significantly exceeding statutory limit values amounted to 25, 2, 200 and 500 mg kg^−1^ for As, Cd, Pb and Zn, respectively (Regulation of the Minister of the Environment, 2016). The highest exceedances over limit values of these elements in soils were recorded in the area of the dump and the electrical and electronic waste processing plant, and the lowest (except Zn) near the A4 motorway. The results indicating high soil contamination in WEEE plants correspond to previous observations regarding pollution from various urban-industrial sources presented in the publication by Albanese and Breward (2011) based on data from Reimann and de Caritat (1998). Albanese and Breward (2011) also found that the electronics industry probably produces the widest range of pollutants and this is reflected in these results. The contents of almost all analyzed elements were the highest in the vicinity of WEEE (except Cd, Mn, Sr and Zn). The high values of these elements in soils sampled in the vicinity of the heap and the highway are the result of their high contents in the geological background (near the heap) as well as, in the case of the highway, vehicle emissions and corrosion of galvanized road infrastructure elements (Wawer et al., 2017).

Table 5 presents the results of calculations of mean geochemical index (Igeo) and pollution load index (PLI). The mean values of Igeo confirm that Cd, Pb and Zn, but also Sb and to a lesser extent also Cu, As, Tl and Mo (in the area of WEEE plant) are the contaminating elements in the studied areas. In the case of soils from the metallurgical dump, the Igeo mean values of Pb, Zn, Cd and Sb are in the 3–5 class, i.e. the soils are moderately polluted or highly polluted to extremely polluted with these elements. In the case of the WEEE plant, the Igeo mean values change in a similar range: from class 3 (moderately polluted—for Zn) to 5 (highly polluted to extremely polluted—for Pb). In case of soil in the area of motorway, the highest Igeo class is 4th class and it concerns Zn. For Pb and Cd class 3 is defined.

**Table 5:**
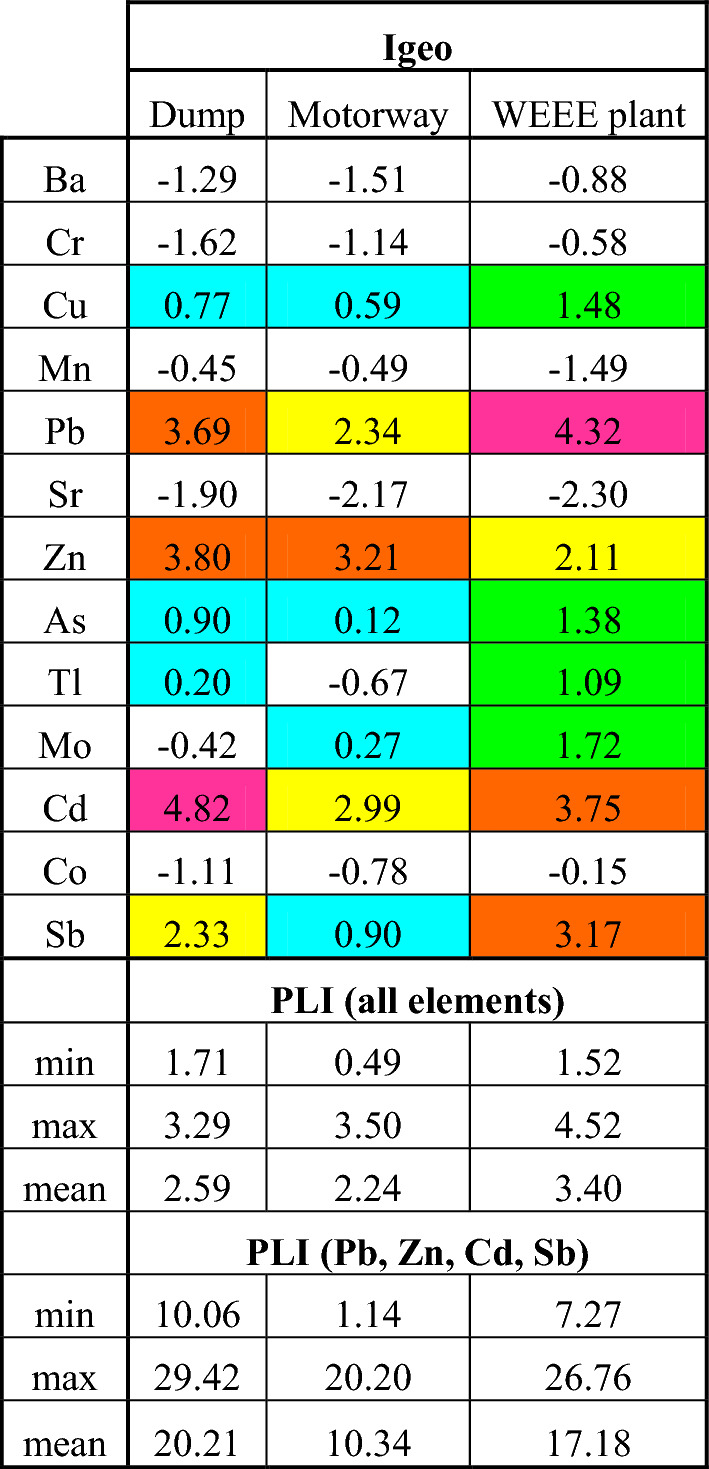
Mean values of the calculated indexes of pollutants

Igeo class

PLI

< 1—excellent soil quality; ~ 1—basic level of soil contamination; > 1—deterioration of soil quality

The PLI index, as a more universal parameter, allows comparison of the studied areas regarding the degree of contamination with a selected group of elements. For the purposes of this study, PLI was calculated in two ways: taking into account all 13 elements in Table 5 and limiting their number to those elements for which mean Igeo was above one in more than one area (i.e. Pb, Zn, Cd, Sb). Regardless of the calculation method, the PLI was always > 1, indicating a deterioration in soil quality. PLI calculated on the basis of all elements oscillated around 2–3 for all investigated areas, while for selected contaminating elements the mean values were: 10.34; 17.18 and 20.21 for the areas of motorway, WEEE plant and metallurgical slag dump, respectively, suggesting extreme contamination.

The Sb/As ratio has been used to identify the source of antimony in soil (Bi et al., 2011; Fu et al., 2011; Sharifi et al., 2016. In the case of the presented study conducted in areas with known dominant sources of elements emission, including antimony, it is possible to accurately assign ranges of values of this ratio to specific sources. This ratio ranged from 0.08 to 0.58 for the dump area, 0.23–0.49 for the impact of the electrical and electronic waste treatment plant and 0.08–0.25 for the motorway area. Similar and higher ranges of this parameter for the areas of the e-waste plant and metallurgical dump indicate that these both objects are the dominant source of antimony. This is consistent with the literature data stating that the dominant source of this element is mining and metallurgy of metal ores (He et al., 2019), including antimony itself (Herath et al., 2017), as well as the burning of fossil fuels and wear of brake blocks in cars (Varrica et al., 2013). The obtained results clearly confirmed that the excessive levels of antimony in the soil, many times exceeding the level of the geochemical background, are of anthropogenic origin in all the studied areas. Taking into account that the research was conducted in the highly industrialized Upper Silesia, it must be stated that in the case of the studied areas mixed sources of antimony and other elements in the soil occur. Both, in the WEEE plant and dump areas, transport emissions from adjacent roads had a significant impact on the soil contamination.

### Soil pollution source apportionment

The results of the factor analysis of samples collected in the region of dump of slag revealed two main sources responsible for around 30 and 40% of the variables (Fig. S2—Supplementary Information). Factor I was responsible mainly for Tl, Mo, Cd, Cu, Mn, Zn, Fe and the other one for Co, Sb, Pb, Sr, Al. Interestingly, both factors determined χ, As, Ba, Cr to a similar degree, which proves that these elements could have had two common sources. These chemical elements are connected with the composition of slag deposited on dump but also very likely to the composition of the sewage sludge. In the research area adjacent to the dump, strong positive Pearson’s correlation coefficients (above 0.70) were obtained for example for Fe and Tl, Cd, Zn, for Sr and Sb, Ba, while Fe–Ba or Sr–Cr correlation was below − 0.70 (Table S3—Supplementary Information). The lack of strong correlations between the greater number of analyzed variables in this region can be explained by the use of sewage sludge as fertilizer in the field, the composition of which can be very diverse. PTEs present in sewage sludge can pose a real threat, especially due to the presence of mobile forms (Feng et al., 2018; Shamuyarira & Gumbo, 2014; Tytła & Widziewicz-Rzońca, 2021).

In the case of samples from the vicinity of WEEE (Fig. S3—Supplementary Information) factor analysis also showed that two factors were mainly responsible for the variables. The first one conditioned about 60% of the variables. Only cadmium and aluminum corresponded to the second and third factors, which may indicate their different origins. High Pearson’s correlation coefficients (over 0.70) between most of the analyzed variables may suggest their common source (Table S4—Supplementary Information). Most likely, it is an electrical waste processing plant.

One factor was responsible for over 70% of soil contamination in the vicinity of the motorway (Fig. S4—Supplementary Information). All associated variables can be derived from road emissions, indicating that this was the main source of potentially toxic elements in the area. Mutual relations and a common source of PTEs confirm strong correlation coefficients between most variables (Table S5—Supplementary Information).

## Conclusions

The results of the research indicate that the soil in the vicinity of the studied objects was characterized by high values of mass-specific magnetic susceptibility (above 50 × 10^–8^ m^3^ kg^−1^), and thus, high contents of PTEs. In all analyzed areas the antimony content was significantly higher than the reported average content of natural origin in soils in Poland, amounting to 0.17 mg kg^−1^. Thus, obtained results clearly confirmed that the excessive levels of antimony in the soil, many times exceeding the level of the geochemical background, are of anthropogenic origin.

The most polluted area was in the vicinity of an electrical waste processing plant. It is true that the values of magnetic susceptibility were lower there than in the other studied areas, but it only proves a lower content of technogenic magnetic particles, i.e. mainly iron oxides and hydroxides. Soil contamination in the vicinity of the WEEE plant was not the result of high-temperature technological processes in which TMPs are formed, but it was the result of mechanical treatment of used electrical and electronic devices. Therefore, the recorded high PTEs content (Sb, As, Cr, Cu and Pb) was not accompanied by high values of magnetic susceptibility. What is more, taking into account all the results of the quantitative analysis of antimony in the tested soil samples, it can be concluded that the WEEE processing and sorting plant has the greatest effect on the antimony content in the soil.

Large amounts of Zn and Cd were found in the soil collected in the vicinity of the heap after the processing of zinc–lead ores. These elements were not only from the emissions of dust and material from the heap, but were also delivered to the soil along with sewage sludge used in the farmlands as fertilizer. The average antimony content was lower than in the vicinity of the area of the electrical and electronic waste processing plant but still very high.

Research has shown that the impact of road traffic and wearing off brake blocks, i.e. traffic anthropopression in general, has little effect on the antimony content in the surrounding soil. Surprisingly, the soil in the vicinity of the highway was the least polluted. The area around WEEE plant was characterized by the following 9 elements with Igeo > 1: Pb > Cd > Sb > Zn > Mo > Cu > As > Tl, the area of metallurgic waste dump area by the following 5 elements with Igeo > 1: Cd > Zn > Pb > Sb, while the area near the highway by the following 4 elements: Zn > Cd > Pb. The Igeo for Pb, Zn and Cd was high in all studied areas, which may indicate that these elements can be emitted by all considered pollution sources.

The research confirmed that thanks to the use of soil magnetometry, it was possible to quickly identify places with a potentially increased content of PTE. Magnetic susceptibility measurements combined with chemical analysis and calculations of pollution indices provided sufficient data for a comprehensive assessment of the condition and degree of soil contamination in the study area. Similar research conducted in the future in other industrial areas with an equally complex impact of emitters will allow the development of specific operating patterns, allowing for a faster assessment of the condition of problem areas and the identification of areas that may pose a potential threat to the local population.

### Supplementary Information

Below is the link to the electronic supplementary material.

Supplementary file1 (DOCX 673 KB)

## Data Availability

Samples of the compounds are available from the authors.

## References

[CR1] Albanese, S., & Breward, N. (2011). Sources of anthropogenic contaminants in the urban environment. In C. C. Johnson, A. Demetriadesm, J. Locutura, & R. T. Oteesen (Eds.), *Mapping the chemical environment of urban areas* (pp. 116–127). Wiley-Blackwell.

[CR2] Ashley, P. M., Graham, B., Tighe, M., & Wolfenden, B. J. (2007). Antimony and arsenic dispersion in the Macleay River catchment, New South Wales, Australia: A study of the environmental geochemical consequences. *Australian Journal of Earth Sciences,**54*, 83–103. 10.1080/08120090600981467 10.1080/08120090600981467

[CR3] Azevedo, L. P., da Silva Araújo, F. G., Lagarinhos, C. A. F., Tenório, J. A. S., & Espinosa, D. C. R. (2017). E-waste management and sustainability: A case study in Brazil. *Environmental Science and Pollution Research,**24*, 25221–25232. 10.1007/s11356-017-0099-7 28929286 10.1007/s11356-017-0099-7

[CR4] Basel Convention. (2005). *Basel convention on the transboundary movements of hazardous wastes and their disposal*. http://www.basel.int/2005 7697032

[CR5] Bhattacharya, P., Jacks, G., Frisbie, S. H., Naidu, R., Smith, E., & Sarkar, B. (2002). Arsenic in the environment: A global perspective. In B. Sarkar (Ed.), *Heavy metals in the environment* (pp. 147–215). Marcel Dekker.

[CR6] Bi, X., Li, Z., Zhuang, X., Han, Z., & Yang, W. (2011). High levels of antimony in dust from e-waste recycling in southeastern China. *Science of the Total Environment,**409*, 5126–5128. 10.1016/j.scitotenv.2011.08.009 21907394 10.1016/j.scitotenv.2011.08.009

[CR7] Blaha, U., Appel, E., & Stanjek, H. (2008). Determination of anthropogenic boundary depth in industrially polluted soil and semi-quantification of heavy metal loads using magnetic susceptibility. *Environmental Pollution,**156*(2), 278–289. 10.1016/j.envpol.2008.02.013 18538906 10.1016/j.envpol.2008.02.013

[CR8] Bolan, N., Kumar, M., Singh, E., Kumar, A., Singh, L., Kumar, S., Keerthanan, S., Hoang, S. A., El-Nagar, A., Vithanage, M., Sarkar, B., Wijesekara, H., Diyabalanage, S., Sooriyakumar, P., Vinu, A., Wang, H., Kirkham, M. B., Shaheen, S. M., Rinklebe, J., & Siddique, H. M. (2022). Antimony contamination and its risk management in complex environmental settings: A review. *Environment International,**158*, 106908. 10.1016/j.envint.2021.106908 34619530 10.1016/j.envint.2021.106908

[CR9] Cabała, J., Żogała, B., & Dubiel, R. (2008). Geochemical and geophysical study of historical Zn–Pb ore processing waste dump areas (southern Poland). *Polish Journal of Environmental Studies,**17*(5), 693–700.

[CR10] Catinon, M., Ayrault, S., Boudouma, O., Bordier, L., Agnello, G., Reynaud, S., & Tissut, M. (2014). Isolation of technogenic magnetic particles. *Science of the Total Environment,**475*, 39–47. 10.1016/j.scitotenv.2013.12.083 24419285 10.1016/j.scitotenv.2013.12.083

[CR11] Chen, B., Krachler, M., & Shotyk, W. (2003). Determination of antimony in plant and peat samples by hydride generation-atomic fluorescence spectrometry (HG-AFS). *Journal of Analytical Atomic Spectrometry,**18*, 1256–1262. 10.1039/B306597A 10.1039/B306597A

[CR12] Denys, S., Tack, K., Caboche, J., & Delalain, P. (2008). Bioaccessibility, solid phase distribution, and speciation of Sb in soils and in digestive fluids. *Chemosphere,**74*, 711–716. 10.1016/j.chemosphere.2008.09.088 19027930 10.1016/j.chemosphere.2008.09.088

[CR13] Douay, F., Pruvot, C., Roussel, H., Ciesielski, H., Fourrier, H., Proix, N., & Waterlot, C. (2008). Contamination in urban soil in an area of Northern France polluted by dust emissions of two smelters. *Water, Air, & Soil Pollution,**188*, 247–260. 10.1007/s11270-007-9541-7 10.1007/s11270-007-9541-7

[CR14] EU Council (1976) Directive 76/464/EEC of 4 May 1976 on pollution caused by certain dangerous substances discharged into the aquatic environment of the Community. *Official Journal of the European Communities*, *129*, 23-9

[CR15] Feng, J. J., Jia, L., Liu, Q. Z., Chen, X. L., & Cheng, J. P. (2018). Source identification of heavy metals in sewage sludge and the effect of influent characteristics: A case study from China. *Urban Water Journal,**15*(4), 381–387. 10.1080/1573062X.2018.1483525 10.1080/1573062X.2018.1483525

[CR16] Fialová, H., Maier, G., Petrovský, E., Kapička, A., Boyko, T., & Scholger, R. (2006). Magnetic properties of soils from sites with different geological and environmental settings. *Journal of Applied Geophysics,**59*, 273–283. 10.1016/j.jappgeo.2005.10.006 10.1016/j.jappgeo.2005.10.006

[CR17] Filella, M., Belzile, N., & Chen, Y. (2002a). Antimony in the environment: A review focused on natural waters I. *Occurrence. Earth-Science Reviews,**57*, 125–176. 10.1016/S0012-8252(01)00070-8 10.1016/S0012-8252(01)00070-8

[CR18] Filella, M., Belzile, N., & Chen, Y. (2002b). Antimony in the environment: A review focused on natural waters II. *Relevant Solution Chemistry. Earth-Science Reviews,**59*, 265–285. 10.1016/S0012-8252(02)00089-2 10.1016/S0012-8252(02)00089-2

[CR19] Filella, M., Williams, P. A., & Belzile, N. (2009). Antimony in the environment: Knowns and unknowns. *Environmental Chemistry,**6*, 95–105. 10.1071/EN09007 10.1071/EN09007

[CR20] Flynn, H. C., Meharg, A. A., Bowyer, P. K., & Paton, G. I. (2003). Antimony bioavailability in mine soils. *Environmental Pollution,**124*, 93–100. 10.1016/s0269-7491(02)00411-6 12683986 10.1016/s0269-7491(02)00411-6

[CR21] Fu, Z., Wu, F., Mo, Ch., Liu, B., Zhu, J., Deng, Q., Liao, H., & Zhang, Y. (2011). Bioaccumulation of antimony, arsenic, and mercury in the vicinities of a large antimony mine, China. *Microchemical Journal,**97*, 12–19. 10.1016/j.microc.2010.06.004 10.1016/j.microc.2010.06.004

[CR22] Gałkiewicz, T., & Śliwiński, S. (1983). Geological characteristic of the Silesian–Cracovian lead–zinc ore deposits. *Annales Societatis Geologorum Poloniae,**53*(1–4), 63–90. (in Polish).

[CR23] Górecka, E. (1993). Geological setting of the Silesian–Cracow Zn–Pb deposits. *Geological Quarterly,**37*(2), 127–145.

[CR24] Guo, W., Fu, Z., Wang, H., Song, F., Wu, F., & Giesy, J. P. (2018). Environmental geochemical and spatial/temporal behavior of total and speciation of antimony in typical contaminated aquatic environment from Xikuangshan, China. *Microchemical Journal,**137*, 181–189. 10.1016/j.microc.2017.10.010 10.1016/j.microc.2017.10.010

[CR25] He, M., Wang, N., Long, X., Zhang, C., Ma, C., Zhong, Q., Wang, A., Wang, Y., Pervaiz, A., & Shan, J. (2019). Antimony speciation in the environment: Recent advances in understanding the biogeochemical processes and ecological effects. *Journal of Environmental Sciences,**75*, 14–39. 10.1016/j.jes.2018.05.023 10.1016/j.jes.2018.05.023 30473279

[CR26] Herath, I., Vithanage, M., & Bundschuh, J. (2017). Antimony as a global dilemma: Geochemistry, mobility, fate and transport. *Environmental Pollution,**223*, 545–559. 10.1016/j.envpol.2017.01.057 28190688 10.1016/j.envpol.2017.01.057

[CR27] Hjortenkrans, D. S. T., Bergbäck, B. G., & Häggerud, A. V. (2007). Metal emissions from brake linings and tires: Case studies of Stockholm, Sweden 1995/1998 and 2005. *Environmental Science & Technology,**41*(15), 5224–5230. 10.1021/es070198o 17822083 10.1021/es070198o

[CR28] ISO 21226:2019. (2019). *International ISO Standard: Soil Quality—Guideline for the Screening of Soil Polluted with Toxic Elements Using Soil Magnetometry*^*,*^* 1st ed*.; International Organization for Standardization: Geneva, Switzerland

[CR29] Jabłońska-Czapla, M., Rosik-Dulewska, C., Szopa, S., & Zerzucha, P. (2015). Research into the metal/metalloid movements in soil and groundwater in the areas surrounding the coal waste dump Hałda Ruda (Upper Silesia, Poland). *Annual Set the Environment Protection,**17*, 367–395.

[CR30] Jones, K. C., Lepp, N. W., & Obbard, J. P. (1990). Other metals and metalloids. In B. J. Alloway (Ed.), *Heavy metals in soils* (p. 280). Blackie, Glasgow.

[CR31] Jordanova, N. (2016). *Soil magnetism. Applications in pedology, environmental science and agriculture* (1st ed.). Academic Press.

[CR32] Kabata-Pendias, A. & Pendias, H. (1999). Biogeochemistry of Trace Elements, 2nd ed., Wyd. Nauk PWN, Warsaw, 400 (Po).

[CR33] Kennedy, P. (2003a). *Metals in particulate material on road surface*. Prepared by Kingett Mitchell Ltd for Ministry of Transport, New Zealand, Infrastructure Auckland.

[CR34] Kennedy, P. (2003b). *Emission factors for contaminants released by motor vehicles in New Zealand*. Prepared by Kingett Mitchell Ltd for Ministry of Transport, New Zealand, Infrastructure Auckland.

[CR35] King, L. D. (1988). Retention of metals by several soils of the southeastern United States. *Journal of Environmental Quality,**17*, 239–246. 10.2134/jeq1988.00472425001700020013x

[CR36] Kondracki, J. (2002). *Geografia regionalna Polski*. PWN. (in Polish).

[CR37] Kulka, E., & Gzy, J. (2008). Assessment of lead and cadmium soil contamination in the vicinity of a non-ferrous metal smelter. *Archives of Environmental Protection,**34*, 105–115.

[CR38] Kumar, A., Holuszko, M., & Espinosa, D. C. R. (2017). E-Waste: An overview on generation, collection, legislation and recycling practices. *Resources, Conservation and Recycling,**122*, 32–42. 10.1016/j.resconrec.2017.01.018 10.1016/j.resconrec.2017.01.018

[CR39] Larner, B. L., Seen, A. J., & Townsend, A. T. (2006). Comparative study of optimized BCR sequential extraction scheme and acid leaching of elements in the certified reference material NIST 2711. *Analytica Chimica Acta,**556*(2), 444–449. 10.1016/j.aca.2005.09.058 10.1016/j.aca.2005.09.058

[CR40] Leita, L., De Nobili, M., Mondini, C., Muhlbachova, G., Marchiol, L., Bragato, G., & Contin, M. (1999). Influence of inorganic and organic fertilization on soil microbial biomass, metabolic quotient and heavy metal bioavailability. *Biology and Fertility of Soils,**28*, 371–376. 10.1007/s003740050506 10.1007/s003740050506

[CR41] Loska, K., Wierchuła, D., & Korus, I. (2004). Antimony concentration in farming soil of southern Poland. *Bulletin of Environmental Contamination and Toxicology,**72*, 858–865. 10.1007/s00128-004-0323-2 15200004 10.1007/s00128-004-0323-2

[CR42] Łukasik, A., Szuszkiewicz, M., & Magiera, T. (2015). Impact of artifacts on topsoil magnetic susceptibility enhancement in urban parks of the Upper Silesian Conurbation datasets. *Journal of Soils and Sediments,**15*, 1836–1846. 10.1007/s11368-014-0966-5 10.1007/s11368-014-0966-5

[CR43] Magiera, T., Gołuchowska, B., & Jabłońska, M. (2013). Technogenic magnetic particles in alkaline dusts from power and cement plants. *Water, Air, & Soil Pollution,**224*, 1389. 10.1007/s11270-012-1389-9 23325986 10.1007/s11270-012-1389-9 PMC3543769

[CR44] Magiera, T., Jabłońska, M., Strzyszcz, Z., & Rachwał, M. (2011). Morphological and mineralogical forms of technogenic magnetic particles in industrial dusts. *Atmospheric Environment,**45*, 4281–4290. 10.1016/j.atmosenv.2011.04.076 10.1016/j.atmosenv.2011.04.076

[CR45] Magiera, T., Strzyszcz, Z., Kapicka, A., & Petrovsky, E. (2006). Discrimination of lithogenic and anthropogenic influences on topsoil magnetic susceptibility in Central Europe. *Geoderma,**130*, 299–311. 10.1016/j.geoderma.2005.02.002 10.1016/j.geoderma.2005.02.002

[CR46] Manaka, M. (2006). Amount of amorphous materials in relationship to arsenic, antimony and bismuth concentrations in a brown forest soil. *Geoderma,**136*, 75–86. 10.1016/j.geoderma.2006.02.002 10.1016/j.geoderma.2006.02.002

[CR47] Martinez, A. M., & Escheberria, J. (2016). Towards a better understanding of the reaction between metal powders and the solid lubricant Sb_2_S_3_ in a low-metallic brake pad at high temperature. *Wear,**348–349*, 27–42. 10.1016/j.wear.2015.11.014 10.1016/j.wear.2015.11.014

[CR48] Mitsunobu, S., Takahashi, Y., & Sakai, Y. (2008). Abiotic reduction of antimony (V) by green rust (Fe(II)(OH)_12_SO_4_·3H_2_O). *Chemosphere,**70*, 942–947. 10.1016/j.chemosphere.2007.07.021 17761212 10.1016/j.chemosphere.2007.07.021

[CR49] Müller, G. (1969). Index of geoaccumulation in sediments of the Rhine River. *The Journal of Geology,**2*, 108–118.

[CR50] Müller, G. (1979). Schwermetalle in den sedimenten des Rheins, Veranderungen Seit 1971. *Umschau in Wissenschaft und Technik,**79*(24), 778–783.

[CR51] Pasieczna, A. (2012). The content of antimony and bismuth in the soils of agricultural lands in Poland. *Polish Journal of Agronomy,**10*, 21–29. (in Polish).

[CR52] Perkins, D. N., Drisse, M. N., Nxele, T., & Sly, P. D. (2014). E-waste: A global hazard. *Annals of Global Health,**80*, 286–295. 10.1016/j.aogh.2014.10.001 25459330 10.1016/j.aogh.2014.10.001

[CR53] Plant, J. A., & Rainswell, R. (1983). Principles of environmental geochemistry. Academic press geology series In I. Thornton (Ed.), *Applied environmental geochemistry. *Academic Press London.

[CR54] PN-ISO 10390:1997-Soil quality-Determination of pH

[CR55] Qi, C., Liu, G., Kang, Y., Lam, P. K. S., & Chou, C. (2011). Assessment and distribution of antimony in soils around three coal mines. Anhui China. *Journal of the Air & Waste Management Association,**61*, 850–857. 10.3155/1047-3289.61.8.850 21874956 10.3155/1047-3289.61.8.850

[CR56] Quan, S. X., Yan, B., Yang, F., Li, N., Xiao, X. M., & Fu, J. M. (2015). Spatial distribution of heavy metal contamination in soils near a primitive e-waste recycling site. *Environmental Science and Pollution Research,**22*, 1290–1298. 10.1007/s11356-014-3420-8 25138553 10.1007/s11356-014-3420-8

[CR57] Rachwał, M., Kardel, K., Magiera, T., & Bens, O. (2017a). Application of magnetic susceptibility in assessment of heavy metal contamination of Saxonian soil (Germany) caused by industrial dust deposition. *Geoderma,**295*, 10–21. 10.1016/j.geoderma.2017.02.007 10.1016/j.geoderma.2017.02.007

[CR58] Rachwał, M., Magiera, T., & Wawer, M. (2015). Coke industry and steel metallurgy as the source of soil contamination by technogenic magnetic particles, heavy metals and polycyclic aromatic hydrocarbons. *Chemosphere,**138*, 863–873. 10.1016/j.chemosphere.2014.11.077 25576132 10.1016/j.chemosphere.2014.11.077

[CR59] Rachwał, M., Wawer, M., Magiera, T., & Steinnes, E. (2017b). Integration of soil magnetometry and geochemistry for assessment of human health risk from metallurgical slag dumps. *Environmental Science and Pollution Research,**24*, 26410–26423. 10.1007/s11356-017-0218-5 28948429 10.1007/s11356-017-0218-5 PMC5719803

[CR60] Regulation of the Minister of Environment of September 1, 2016 on the method of assessing pollution of the earth’s surface.

[CR61] Reimann, C., & de Caritat, P. (1998). *Chemical elements in the environment*. Springer Verlag.

[CR62] Rożek, D., Nadłonek, W., & Cabała, J. (2015). Forms of heavy metals (Zn, Pb, Cd) occurring in rhizospheres from the areas of former and contemporary Zn–Pb ore mining. *Mining Science,**22*, 125–138. 10.5277/ms150210 10.5277/ms150210

[CR63] Seńczuk, W. (1990). Toxicology. Państwowy Zakład Wydawnictw Lekarskich, Warszawa. (in Polish)

[CR64] Shamuyarira, K. K., & Gumbo, J. R. (2014). Assessment of heavy metals in municipal sewage sludge: A case study of Limpopo Province, South Africa. *International Journal of Environmental Research and Public Health,**11*(3), 2569–2579. 10.3390/ijerph110302569 24595211 10.3390/ijerph110302569 PMC3986993

[CR65] Sharifi, R., Moore, F., & Keshavarzi, B. (2016). Mobility and chemical fate of arsenic and antimony in water and sediments of Sarouq River cachment, Takab geothermal field, northwest Iran. *Journal of Environmental Management,**170*, 136–144. 10.1016/j.jenvman.2016.01.018 26820974 10.1016/j.jenvman.2016.01.018

[CR66] Singh, R. P., & Agrawal, M. (2008). Potential benefits and risks of land application of sewage sludge. *Waste Management,**28*, 347–358. 10.1016/j.wasman.2006.12.010 17320368 10.1016/j.wasman.2006.12.010

[CR67] Stępniewska, Z., Ostrowski, J., Stępniewski, W., & Gliński, J. (2004). Klasyfikacja odporności oksydoredukcyjnej gleb ornych Polski i ich przestrzenna charakterystyka. *Water-Environment-Rural Areas,**4*(2), 125–133. (in Polish).

[CR68] Świetlik, R., Strzelecka, M., & Trojanowska, M. (2013). Evaluation of traffic-related heavy metals emissions using noise barrier road dust analysis. *Polish Journal of Environmental Studies,**22*(2), 561–567.

[CR69] Telford, K., Maher, W., Krikowa, F., & Foster, S. (2008). Measurement of total antimony and antimony species in mine contaminated soils by ICP-MS and HPLC-ICP-MS. *Journal of Environmental Monitoring,**10*(1), 136–140. 10.1039/b715465h 18175027 10.1039/b715465h

[CR70] Thorpe, A., & Harrison, R. M. (2008). Sources and properties of non-exhaust particulate matter from road traffic: A review. *Science of the Total Environment,**400*, 270–282. 10.1016/j.scitotenv.2008.06.007 18635248 10.1016/j.scitotenv.2008.06.007

[CR71] Tighe, M., & Lockwood, P. (2007). The importance of non-crystalline hydroxide phases in sequential extractions to fractionate antimony in acid soils. *Communications in Soil Science and Plant Analysis,**38*, 1487–1501. 10.1080/00103620701378441 10.1080/00103620701378441

[CR72] Tokarska-Guzik, B., Rostański. A., & Kupka R. (2002) *Katowice. Przyroda miasta, Katowice: Wydawnictwo Kubajak*. ISBN 83-87971-49-9. (in Polish)

[CR73] Tomlinson, D. L., Wilson, J. G., Haris, C. R., & Jefrey, D. W. (1980). Problems in the assessment of heavy metal level in estuaries and the formation of a pollution index. *Helgolander Meeresuntersuchungen,**33*, 566–575. 10.1007/BF02414780 10.1007/BF02414780

[CR74] Tschan, M., Robinson, B. H., & Schulin, R. (2009). Antimony in the soil-plant system—A review. *Environmental Chemistry,**6*, 106–115. 10.1071/EN08111 10.1071/EN08111

[CR75] Tytła, M., & Widziewicz-Rzońca, K. (2021). Heavy metals in municipal sewage sludge—A brief characteristic of potential threats and methods used to assess the ecological risk. *Environment, Earth, and Ecology,**5*(1), 18–25. 10.24051/eee/134276 10.24051/eee/134276

[CR76] Ullrich, S. M., Ramsey, M. H., & Helios-Rybicka, E. (1999). Total and exchangeable concentrations of heavy metals in soils near Bytom, an area of Pb/Zn mining and smelting in Upper Silesia, Poland. *Applied Geochemistry,**14*, 187–196. 10.1016/S0883-2927(98)00042-0 10.1016/S0883-2927(98)00042-0

[CR77] Urionabarrenetxea, E., Casás, C., Garcia-Velasco, N., Santos, M., Tarazona, J. V., & Soto, M. (2022). Predicting environmental concentrations and the potential risk of Plant Protection Products (PPP) on non-target soil organisms accounting for regional and landscape ecological variability in European soils. *Chemosphere,**303*, 135045. 10.1016/j.chemosphere.2022.135045 35609662 10.1016/j.chemosphere.2022.135045

[CR78] Varrica, D., Bardelli, F., Dongarrà, G., & Tamburo, E. (2013). Speciation of Sb in airborne particulate matter, vehicle brake linings, and brake pad wear residues. *Atmospheric Environment,**64*, 18–24. 10.1016/j.atmosenv.2012.08.067 10.1016/j.atmosenv.2012.08.067

[CR79] de Vos, W.D., Tarvainen, T., Salminen, R., Reeder, S., Vivo, B.D., Demetriades, A., Pirc, S., Batista, M.J., Marsina, K., Ottesen, R.T., O'Connor, P., Bidovec, M., Lima, A., Siewers, U., Smith, B., Taylor, H., Shaw, R., Salpeteur, I., Gregorauskienė, V., Halamić, J., Slaninka, I., Lax, K., Gravesen, P., Birke, M., Breward, N., Ander, E.L., Jordan, G., Ďuriš, M., Klein, P., Locutura, J., Bel-lan, A., Pasieczna, A., Lis, J., Mazreku, A., Gilucis, A., Heitzmann, P., Klaver, G.T., & Petersell, V. (2006). *Geochemical atlas of Europe. Part 2, Interpretation of geochemical maps, additional tables, figures, maps, and related publications*.

[CR80] Warchulski, R., Gawęda, A., Kądziołka-Gaweł, M., & Szopa, K. (2015). Composition and element mobilization in pyrometallurgical slags from the Orzeł Biały smelting plant in the Bytom Piekary Śląskie area. *Poland. Mineralogical Magazine,**79*(2), 459–483. 10.1180/minmag.2015.079.2.21 10.1180/minmag.2015.079.2.21

[CR81] Wawer, M., Magiera, T., Ojha, G., Appel, E., Kusza, G., Hu, S., & Basavaiah, N. (2015). Traffic-related pollutants in roadside soils of different countries in Europe and Asia. *Water, Air, & Soil Pollution,**226*(7), 1–14. 10.1007/s11270-015-2483-6 10.1007/s11270-015-2483-6

[CR82] Wawer, M., Rachwał, M., & Kowalska, J. (2017). Impact of noise barriers on the dispersal of solid pollutants from car emissions and their deposition in soil. *Soil Science Annual,**68*(1), 19–26. 10.1515/ssa-2017-0003 10.1515/ssa-2017-0003

[CR83] Wedepohl, K. H. (Eds.) (1969–1974). *Handbook of geochemistry*. Springer-Verlag (several volumes).

[CR84] Wilson, S. C., Lockwood, P. V., Ashley, P. M., & Tighe, M. (2010). The chemistry and behaviour of antimony in the soil environment with comparisons to arsenic: A critical review. *Environmental Pollution,**158*, 1169–1181. 10.1016/j.envpol.2009.10.045 19914753 10.1016/j.envpol.2009.10.045

[CR85] Yang, H., & He, M. (2015). Speciation of antimony in soil and sediments by liquid chromatography–hydride generation–atomic fluorescence spectrometry. *Analytical Letters,**48*, 1941–1953. 10.1080/00032719.2015.1004077 10.1080/00032719.2015.1004077

[CR86] Yurddaskal, M., Nil, M., Ozturk, Y., & Celik, E. (2018). Synergetic effect of antimony trioxide on the flame retardant and mechanical properties of polymer composites for consumer electronics applications. *Journal of Materials Science: Materials in Electronics,**29*(6), 4557–4563. 10.1007/s10854-017-8405-1 10.1007/s10854-017-8405-1

[CR87] Zeng, X., Wang, F., Li, J., & Gong, R. (2017). A simplified method to evaluate the recycling potential of e-waste. *Journal of Cleaner Production,**168*, 1518–1524. 10.1016/j.jclepro.2017.06.232 10.1016/j.jclepro.2017.06.232

