# Supplementary material for: Application of soil magnetometry and geochemical methods to investigate soil contamination with antimony
Source: Environ Geochem Health. 2024 Jul 6;46(8):287. doi: 10.1007/s10653-024-02086-0 (PMC11227468; doi:10.1007/s10653-024-02086-0)
Supplement: Supplementary file 1 — Supplementary file1 (DOCX 673 KB) [file 10653_2024_2086_MOESM1_ESM.docx]

**Supplementary Information**

**Application of soil magnetometry and geochemical methods to investigate soil contamination with antimony**

Magdalena Jabłońska-Czapla^1^, Marzena Rachwał^1,2^, Katarzyna Grygoyć^1^, Małgorzata Wawer-Liszka^1*^

^1^Institute of Environmental Engineering of the Polish Academy of Sciences,

34 M. Skłodowska-Curie Street, 41-819, Zabrze, Poland;

^2^Institute of Safety Engineering, The Main School of Fire Service, 52/54 Slowackiego St.,

01-629, Warsaw, Poland

**Fig. S1** Sequential chemical extraction of antimony (BCR method); F0 mobile fraction dissolved in water, F1 mobile exchangeable fraction associated with adsorbed cations and anions, carbonates, and very reactive oxy-hydroxides; F2 mobile reducible fraction associated with iron/manganese oxides; F3 immobile oxidizable fraction associated with organic substance and sulfides; R immobile residual fraction associated with non-silicate bound metals

**Fig.S2** Results of factor analysis of soil samples from the dump area

**Fig. S3** Results of factor analysis of soil samples from the electrical and electronic waste processing plant

**Fig. S4** Results of factor analysis of soil samples from the highway region

Table S1 Operating parameters of the ICP-MS and ICP-OES spectrometer

| Parameter | Value |
| --- | --- |
| ICP-MS | |
| RF power [W] | 1125 |
| Plasma gas ﬂow [L/min] | 15 |
| Nebulizer gas ﬂow [L/min] | 0.76–0.82 |
| Auxiliary gas ﬂow [L/min] | 1.15–1.16 |
| Nebulizer type | Cross ﬂow |
| Plasma torch | Quartz |
| Scanning mode | Peak hopping |
| Dwell time [ms] | 100 |
| Sweeps/reading | 20 |
| Number of replicates | 3 |
| ICP-OES | |
| RF power [W] | 1400 |
| Plasma gas ﬂow [L/min] | 10 |
| Nebulizer gas ﬂow [L/min] | 0.6 |
| Auxiliary gas ﬂow [L/min] | 0.2 |
| Sample uptake rate [mL/min] | 1 |
| Nebulizer type | Cross ﬂow |
| Spray chamber | Scott’s type |
| Plasma torch | Quartz |
| Scanning mode | Peak area |
| Number of replicates | 3 |

Table S2 Physicochemical parameters of soils samples

| **Sampling points** | **pH in H_2_O** | **pH in KCl** | **Eh** |
| --- | --- | --- | --- |
| **Dump area Piekary** | | | |
| P01 | 6.48 | 6.46 | 183.0 |
| P02 | 6.56 | 6.52 | 230.0 |
| P06 | 6.09 | 5.96 | 293.1 |
| P11 | 7.12 | 7.07 | 245.6 |
| P12 | 6.28 | 6.23 | 258.0 |
| P17 | 7.37 | 7.34 | 229.5 |
| **WEEE plant Katowice** | | | |
| E05 | 3.83 | 3.49 | 349.3 |
| E09 | 3.85 | 3.59 | 384.5 |
| E10 | 3.43 | 3.14 | 425.5 |
| E22 | 4.33 | 3.68 | 376.2 |
| E25 | 3.62 | 3.46 | 375.8 |
| E27 | 3.35 | 3.11 | 398.5 |
| **HighwayA4 area Katowice** | | | |
| A04 | 6.89 | 6.84 | 262.3 |
| A05 | 6.81 | 6.74 | 267.5 |
| A06 | 6.66 | 6.59 | 262.1 |
| A07 | 6.67 | 6.63 | 255.3 |
| A09 | 6.69 | 6.64 | 256.4 |
| A11 | 6.52 | 6.48 | 254.5 |

Table S3 Pearson's correlation matrix between the analyzed elements in soils from the dump area

| Variable | Mean  Conc.  [mg^.^kg^-1^] | SD. | χ | As | Tl | Mo | Cd | Co | Sb | Ba | Cr | Cu | Mn | Pb | Sr | Zn | Al | Fe |
| --- | --- | --- | --- | --- | --- | --- | --- | --- | --- | --- | --- | --- | --- | --- | --- | --- | --- | --- |
| χ | 211.94 | 82.74 | 1.00 |  |  |  |  |  |  |  |  |  |  |  |  |  |  |  |
| As | 38.38 | 21.45 | 0.68 | 1.00 |  |  |  |  |  |  |  |  |  |  |  |  |  |  |
| Tl | 1.50 | 0.51 | 0.27 | 0.47 | 1.00 |  |  |  |  |  |  |  |  |  |  |  |  |  |
| Mo | 1.10 | 0.32 | 0.14 | 0.20 | 0.41 | 1.00 |  |  |  |  |  |  |  |  |  |  |  |  |
| Cd | 13.69 | 7.27 | 0.20 | 0.42 | 0.83 | 0.16 | 1.00 |  |  |  |  |  |  |  |  |  |  |  |
| Co | 7.40 | 1.63 | 0.47 | 0.79 | 0.39 | 0.67 | 0.11 | 1.00 |  |  |  |  |  |  |  |  |  |  |
| Sb | **11.31** | **10.81** | **0.23** | **0.28** | **-0.01** | **-0.07** | **-0.47** | **0.40** | **1.00** |  |  |  |  |  |  |  |  |  |
| Ba | 262.90 | 107.42 | 0.04 | -0.02 | -0.35 | -0.13 | -0.59 | 0.13 | **0.59** | 1.00 |  |  |  |  |  |  |  |  |
| Cr | 47.39 | 10.61 | 0.09 | -0.07 | 0.54 | 0.38 | 0.65 | -0.10 | **-0.58** | -0.73 | 1.00 |  |  |  |  |  |  |  |
| Cu | 51.61 | 28.74 | 0.66 | 0.76 | 0.52 | 0.30 | 0.39 | 0.63 | **0.25** | -0.30 | 0.39 | 1.00 |  |  |  |  |  |  |
| Mn | 600.54 | 175.52 | 0.34 | 0.33 | 0.61 | 0.68 | 0.45 | 0.49 | **-0.12** | -0.26 | 0.25 | 0.15 | 1.00 |  |  |  |  |  |
| Pb | 700.55 | 323.39 | 0.50 | 0.57 | 0.47 | 0.13 | 0.03 | 0.57 | **0.83** | 0.39 | -0.17 | 0.61 | 0.11 | 1.00 |  |  |  |  |
| Sr | 62.55 | 53.,41 | 0.13 | 0.28 | -0.20 | -0.01 | -0.53 | 0.45 | **0.78** | 0.91 | -0.79 | -0.05 | -0.13 | 0.60 | 1.00 |  |  |  |
| Zn | 1525.90 | 570.72 | 0.55 | 0.69 | 0.90 | 0.29 | 0.88 | 0.44 | **-0.10** | -0.32 | 0.52 | 0.67 | 0.52 | 0.44 | -0.19 | 1.00 |  |  |
| Al | 11.41 | 4.72 | -0.57 | -0.56 | 0.28 | 0.20 | 0.33 | -0.43 | **-0.51** | -0.59 | 0.75 | -0.11 | -0.03 | -0.38 | -0.70 | 0.03 | 1.00 |  |
| Fe | 18.72 | 5.17 | 0.53 | 0.47 | 0.72 | 0.45 | 0.76 | 0.34 | **-0.33** | -0.74 | 0.72 | 0.64 | 0.67 | 0.10 | -0.59 | 0.77 | 0.22 | 1.00 |

χ – susceptibility; SD – Standard Deviation

Table S4 Pearson's correlation matrix between the analyzed elements in soils from the area of the electrical and electronic waste processing plant

| Variable | Mean  Conc.  [mg^.^kg^-1^] | SD. | χ | As | Tl | Mo | Cd | Co | Sb | Ba | Cr | Cu | Mn | Pb | Sr | Zn | Al | Fe |
| --- | --- | --- | --- | --- | --- | --- | --- | --- | --- | --- | --- | --- | --- | --- | --- | --- | --- | --- |
| χ | 680.98 | 190.40 | 1.00 |  |  |  |  |  |  |  |  |  |  |  |  |  |  |  |
| As | 52.13 | 16.88 | 0.90 | 1.00 |  |  |  |  |  |  |  |  |  |  |  |  |  |  |
| Tl | 2.75 | 0.57 | 0.66 | 0.69 | 1.00 |  |  |  |  |  |  |  |  |  |  |  |  |  |
| Mo | 5.11 | 1.66 | 0.82 | 0.76 | 0.27 | 1.00 |  |  |  |  |  |  |  |  |  |  |  |  |
| Cd | 8.72 | 11.10 | 0.23 | 0.24 | -0.32 | 0.70 | 1.00 |  |  |  |  |  |  |  |  |  |  |  |
| Co | 15.72 | 4.92 | 0.91 | 0.80 | 0.44 | 0.80 | 0.21 | 1.00 |  |  |  |  |  |  |  |  |  |  |
| Sb | **16.30** | **6.05** | **0.76** | **0.82** | **0.52** | **0.74** | **0.19** | **0.80** | **1.00** |  |  |  |  |  |  |  |  |  |
| Ba | 357.54 | 120.35 | 0.45 | 0.57 | 0.47 | 0.30 | -0.31 | 0.53 | **0.72** | 1.00 |  |  |  |  |  |  |  |  |
| Cr | 102.32 | 27.56 | 0.84 | 0.75 | 0.17 | 0.89 | 0.52 | 0.90 | **0.71** | 0.37 | 1.00 |  |  |  |  |  |  |  |
| Cu | 82.18 | 29.42 | 0.83 | 0.85 | 0.46 | 0.89 | 0.49 | 0.72 | **0.73** | 0.54 | 0.81 | 1.00 |  |  |  |  |  |  |
| Mn | 344.63 | 173.07 | 0.50 | 0.66 | 0.39 | 0.31 | -0.10 | 0.52 | **0.73** | 0.59 | 0.51 | 0.42 | 1.00 |  |  |  |  |  |
| Pb | 1116.38 | 413.09 | 0.69 | 0.83 | 0.60 | 0.69 | 0.32 | 0.50 | **0.77** | 0.55 | 0.57 | 0.86 | 0.63 | 1.00 |  |  |  |  |
| Sr | 41.13 | 7.58 | 0.58 | 0.52 | 0.62 | 0.30 | -0.39 | 0.55 | **0.66** | 0.88 | 0.37 | 0.54 | 0.50 | 0.56 | 1.00 |  |  |  |
| Zn | 486.66 | 162.43 | 0.75 | 0.79 | 0.86 | 0.44 | -0.08 | 0.49 | **0.62** | 0.49 | 0.42 | 0.66 | 0.65 | 0.85 | 0.65 | 1.00 |  |  |
| Al | 14.94 | 1.18 | 0.21 | 0.03 | 0.32 | 0.21 | 0.11 | -0.09 | **0.04** | 0.00 | -0.01 | 0.34 | -0.21 | 0.41 | 0.36 | 0.43 | 1.00 |  |
| Fe | 31.58 | 7.88 | 0.93 | 0.93 | 0.61 | 0.87 | 0.32 | 0.85 | **0.82** | 0.63 | 0.82 | 0.95 | 0.48 | 0.81 | 0.64 | 0.71 | 0.23 | 1.00 |

χ – susceptibility; SD – Standard Deviation

Table S5 Pearson's correlation matrix between the analyzed elements in soils from the highway region

| Variable | Mean  Conc.  [mg^.^kg^-1^] | SD | χ | As | Tl | Mo | Cd | Co | Sb | Ba | Cr | Cu | Mn | Pb | Sr | Zn | Al | Fe |
| --- | --- | --- | --- | --- | --- | --- | --- | --- | --- | --- | --- | --- | --- | --- | --- | --- | --- | --- |
| χ | 195.71 | 127.82 | 1.00 |  |  |  |  |  |  |  |  |  |  |  |  |  |  |  |
| As | 24.16 | 15.29 | 0.61 | 1.00 |  |  |  |  |  |  |  |  |  |  |  |  |  |  |
| Tl | 0.83 | 0.31 | 0.53 | 0.79 | 1.00 |  |  |  |  |  |  |  |  |  |  |  |  |  |
| Mo | 1.89 | 1.20 | 0.35 | 0.67 | 0.61 | 1.00 |  |  |  |  |  |  |  |  |  |  |  |  |
| Cd | 4.26 | 2.64 | 0.86 | 0.76 | 0.85 | 0.52 | 1.00 |  |  |  |  |  |  |  |  |  |  |  |
| Co | 10.11 | 4.84 | 0.76 | 0.90 | 0.87 | 0.50 | 0.92 | 1.00 |  |  |  |  |  |  |  |  |  |  |
| Sb | **3.88** | **3.18** | **0.68** | **0.85** | **0.62** | **0.24** | **0.68** | **0.83** | **1.00** |  |  |  |  |  |  |  |  |  |
| Ba | 217.98 | 81.66 | 0.82 | 0.55 | 0.67 | 0.25 | 0.90 | 0.74 | **0.65** | 1.00 |  |  |  |  |  |  |  |  |
| Cr | 70.47 | 31.57 | 0.79 | 0.69 | 0.54 | 0.71 | 0.76 | 0.71 | **0.44** | 0.49 | 1.00 |  |  |  |  |  |  |  |
| Cu | 47.27 | 26.52 | 0.69 | 0.96 | 0.73 | 0.53 | 0.79 | 0.94 | **0.87** | 0.56 | 0,.74 | 1.00 |  |  |  |  |  |  |
| Mn | 622.90 | 320.18 | 0.92 | 0.54 | 0.42 | 0.35 | 0.75 | 0.71 | **0.54** | 0.61 | 0.84 | 0.67 | 1.00 |  |  |  |  |  |
| Pb | 375.22 | 270.77 | 0.59 | 0.73 | 0.62 | 0.31 | 0.76 | 0.85 | **0.61** | 0.54 | 0.68 | 0.85 | 0.62 | 1.00 |  |  |  |  |
| Sr | 46.02 | 20,.41 | 0.96 | 0.66 | 0.64 | 0.25 | 0.91 | 0.84 | **0.78** | 0.91 | 0.66 | 0.74 | 0.84 | 0.66 | 1.00 |  |  |  |
| Zn | 1401.80 | 1004.91 | 0.72 | 0.75 | 0.68 | 0.42 | 0.87 | 0,.89 | **0.62** | 0.66 | 0.78 | 0.86 | 0.73 | 0.97 | 0.76 | 1.00 |  |  |
| Al | 13.51 | 4.14 | 0.80 | 0.54 | 0.53 | 0.22 | 0.83 | 0.75 | **0.51** | 0.69 | 0.77 | 0.70 | 0.77 | 0.87 | 0.80 | 0.92 | 1.00 |  |
| Fe | 18.35 | 7.94 | 0.87 | 0.75 | 0.75 | 0.50 | 0.94 | 0.92 | **0.63** | 0.73 | 0.87 | 0.83 | 0.88 | 0.85 | 0.87 | 0.93 | 0.89 | 1.00 |

χ – susceptibility; SD – Standard Deviation
